# Physiological attributes and transcriptomics analyses reveal the mechanism response of *Helictotrichon virescens* to low temperature stress

**DOI:** 10.1186/s12864-022-08526-4

**Published:** 2022-04-07

**Authors:** Mingjun Cheng, Kuoshu Cui, Mingmin Zheng, Tao Yang, Junjun Zheng, Xiaofeng Li, Xuan Luo, Yang Zhou, Ruizhen Zhang, Donghai Yan, Mingjiu Yao, Muhammad Zafar Iqbal, Qingping Zhou, Ruyu He

**Affiliations:** 1 Institute of qinghai-tibetan Plateau, Southwest Minzu University, Chengdu, 610041 China; 2 Sichuan Grass Industry Technology Research and Promotion Center, Chengdu, 610041 China; 3 Sichuan Agricultural Technology Extension Station, Chengdu, 610041 China; 4 College of Chemistry and Life Sciences, Chengdu Normal University, Chengdu, 611130 China; 5 Maize Research Institute, Sichuan Agricultural University, Chengdu, 611130 China; 6 Institute of Agricultural Information and Rural Economy, Sichuan Academy of Agricultural Sciences, Sichuan, Chengdu, 610066 China

**Keywords:** *Helictotrichon virescens*, Low temperature stress, Photosynthesis, Circadian rhythm, LHY, HY5

## Abstract

**Background:**

*Helictotrichon virescens* is a perennial grass that is primarily distributed in high altitude areas of 2000 ~ 4500 m. It is widely cultivated in the Qinghai-Tibet Plateau of China, strongly resistant to cold, and an essential part of the wild herbs in this region. However, the molecular mechanism of the response of *H. virescens* to low temperature stress and the key regulatory genes for specific biological processes are poorly understood.

**Results:**

Physiological and transcriptome analyses were used to study the cold stress response mechanism in *H virescens.* During the low temperature stress period, the content of chlorophyll a and b decreased more and more with the delay of the treatment time. Among them, the difference between the controls was not significant, and the difference between the control and the treatment was significant. At the same time, the expression of related differential genes was up-regulated during low temperature treatment. In addition, the plant circadian pathway is crucial for their response to cold stress. The expression of differentially expressed genes that encode LHY and HY5 were strongly up-regulated during cold stress.

**Conclusions:**

This study should help to fully understand how *H. virescens* responds to low temperatures. It answers pertinent questions in the response of perennial herbs to cold stress, i.e., how light and low temperature signals integrate to regulate plant circadian rhythms and Decrease of content of chlorophylls (which can be also accompanied with decrease of total quantity of reaction centers) leads to an increase in photosynthetic damage.

**Supplementary Information:**

The online version contains supplementary material available at 10.1186/s12864-022-08526-4.

## Background

*Helictotrichon* Bess is a gramineous perennial grass composed of more than 100 species that grows throughout the world. It is primarily distributed in Asia, Europe, North America, and China. More than 20 of the species are an important part of wild forages in China and are distributed in high altitude areas at 2000 ~ 4500 m. Among them, alpine oatgrass (*H. virescens)* is primarily distributed in high altitude areas, such as Tibet and Qinghai. Typically, it is strongly tolerant to cold, can survive winter temperatures as low as − 25 °C and has a life span of approximately 10 years. Its artificial cultivation in the range of 2000-4000 m above sea level can increase the yields of seed and forage with an average annual yield of 0.948 t/hm^2^ seeds per mu and a yield of 7.74 t/hm^2^ of hay. It could be a first choice to improve the natural grasslands, construct artificial grasslands, and green the ecological environment of the Tibetan Plateau. However, the molecular mechanism of the response of this plant to low temperature stress remains unclear, and the key genes that regulate related biological processes are poorly understood.

Low temperature stress is a multi-genic process accompanied by physiological adjustments in the plant. The mechanisms of response of woody species to cold stress are more complicated than those of herbaceous ones [1]. Although much is known about the phenotypic responses of herbs to photoperiod and low temperature, little is known at the molecular level. Low temperatures can affect all aspects of plant physiology and metabolism, including photosynthesis, which is affected the most by low temperature stress [2]. Chlorophyll is the primary pigment for photosynthesis. The first step of photosynthesis is the absorption of light energy by chlorophyll and then its ionization. Both chlorophyll a and b can absorb light energy, but only a few chlorophyll a molecules in the excitation state can convert light energy into electrical energy [3]. During the first step of photosynthesis, antenna proteins capture and transfer light energy and convert it into chemical energy. Most of the energy is absorbed by the light antenna complex (LHCs). The LHC includes most of chlorophyll a and all of chlorophyll b and carotenoids, which can combine with photosystem I (PSI) and photosystem II (PSII) [4, 5]. Previous studies have shown, in nature, there is an interaction between light, water and temperature constraints. Flexible mechanisms are needed to up and down regulate the efficiency of photosynthetic response according to the needs of plant metabolism. This comprehensive regulation is achieved by controlling the excitation energy transfer between the two photosystems (PSII and PSI), the electrochemical gradient across the thylakoid membrane and the electron transfer from PSII to PSI electron acceptor. Stimulation of PSII damage can protects PSI under action of stressors and contributes tolerance of photosynthetic machinery [6]).

The reduction in rate of photosynthesis rate in plant leaves under low temperature conditions is related to the low temperature damage to the PSII reaction centers and chloroplasts. PSII plays an essential role in the responses of plants to low temperature stress [7]. Previous research showed that the ability of plants to absorb and utilize light energy is significantly reduced under adverse conditions, leading to intensified photoinhibition [8].

Under low temperature and low light conditions, the degree of photoinhibition of PSII and PSI intensifies, which is probably closely related to the accumulation of reactive oxygen species (ROS). It is challenging for the plant to recover once PSII has undergone photoinhibition. Typically, it takes several days to recover completely. However, PSII photoinhibition can completely recover within a day or even a few hours. After photoinhibition, the recovery of PSI decreases, which could be the main limiting factor of the decrease in the rate of photosynthesis at low temperatures. After photoinhibition, the PSII activity and light intensity significantly affect the recovery of PSI in vivo [8]. The data suggests that photoinhibition is a temporary or persistent form of light protection and plays an essential role during cold stress. Plants synchronize the endogenous rhythm (physiology and metabolism) with the environmental rhythm by integrating changes with environmental conditions (light and temperature) during the day-night cycle [9]. Many genes have been reported to respond to light and vary regularly during the daylight dark cycle in the manner of a circadian rhythm. For example, E3 ubiquitin-protein ligase COP1 post-transcriptionally affects the expression of HY5 [10], a transcription factor of the basic leucine zipper (bZIP) family that regulates multiple downstream photoreceptors [11, 12]. These genes are transcriptionally affected by light and low temperature and relate to the complete development of cold tolerance in *Arabidopsis thaliana* [13]. LHY is an MYB-related transcription factor categorized as a morning gene of the circadian clock [14]. Studies have also shown that the biological clock is the gateway to induce genes related to cold, including C-repeat Binding Factor (CBF) [15]. The CBF transcription factor plays a key role in low temperature stress in plants and is one of the essential transcription factors that can interact with DRE/CRT *cis* to regulate the gene expression induced by multiple adverse conditions. Proteins encoded by COR (Cold Regulated) genes are strongly hydrophobic and have a high degree of thermal stability. They can play an essential role in preventing the cell dehydration damage caused by low temperature stress. The overexpression of CBF genes in *A. thaliana* [16] or the transfer of CBF homologous genes into other plant species could enhance the tolerance to low temperature stress.

The response mechanism of perennial herbaceous plants to cold stress was studied using *He. virescens* as the research material. The unique biological characteristics of *He. virescens* offer an opportunity to explore the role of light protection and circadian rhythm during cold stress and lay a theoretical foundation to better understand of the response mechanism to cold stress in perennial crops.

## Results

### Morphological and physiological changes in *Helictotrichon virescens* under low temperature stress

**Fig. 1:**
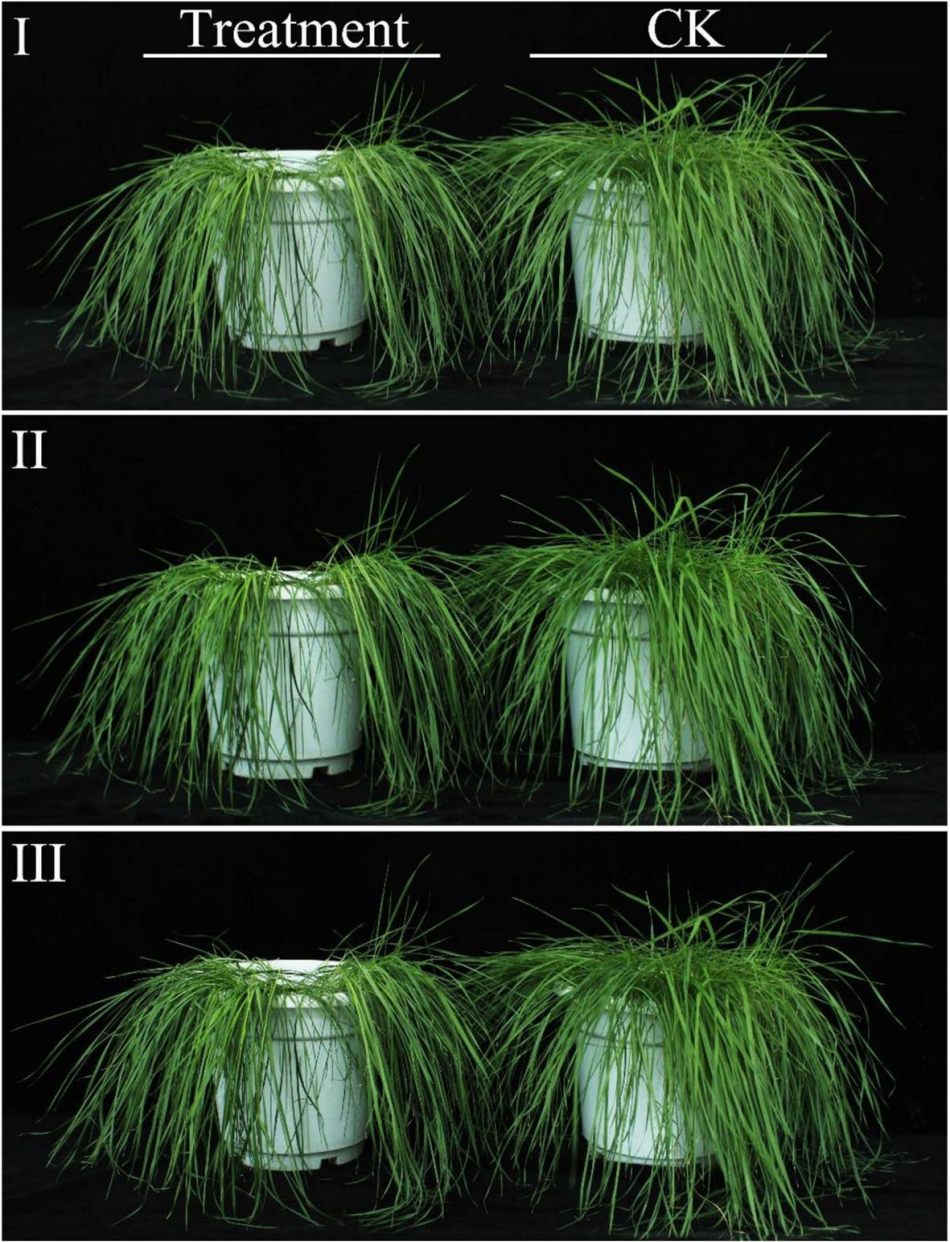
I, II and III show growth conditions of *Helictotrichon Virescens* at low temperature for 12 h, 36 h and 60 h, respectively. Treatment was 0 °C and CK was 25 °C. BLYM: *Helictotrichon virescens*

**Fig. 2:**
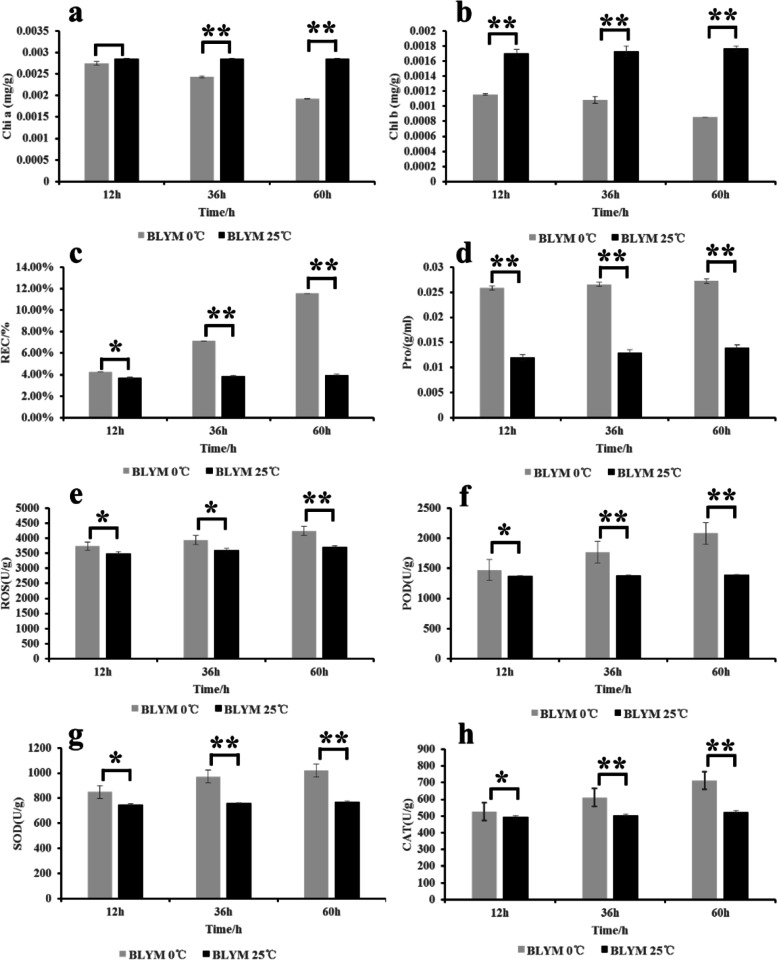
Physiological responses of cold tolerance adaptability of *Helictotrichon Virescens*. BLYM: *Helictotrichon virescens*

Under low temperature stress, the leaves of *He. virescens* wilted and the leaf shrank, which led to water loss (Fig. 1). The chlorophyll content gradually decreased as the time of stress was extended (Fig. 2a and b). After 12 h of low temperature stress, the contents of chlorophyll b were significantly reduced, but the change in chlorophyll a was nonsignificant. After 36 h and 60 h of low temperature stress, the contents of chlorophyll a and b in the treatment group were significantly lower than those in the control group. These results indicated that the content of chlorophyll changed significantly after low temperature stress, and chlorophyll b decreased more rapidly in response to low temperature stress. In addition, the relative conductivity of leaves increased after low temperature stress (Fig. 2c). After 12 h of low temperature stress, the relative conductivity of leaves in the treatment group was significantly higher by 1.15-fold than that of the control group. After 60 h of low temperature stress, the relative EC of leaves in the treatment group increased up to 2.90-fold compared with that of the control group. These results indicated the stability and tolerance of the leaf cell membrane affected by low temperature stress. Under low temperature stress, the accumulation of Pro could reduce the freezing point of cell solutes and improve the osmotic potential, thus, stabilizing the cell membrane system to prevent freezing dehydration and decrease the exudation of solutes. After 12 h of low temperature stress, the Pro content of leaves in the treatment and control groups differed highly significantly (Fig. 2d). The Pro contents in the treatment group were 2.16-fold higher than those of the control group. By extending the stress time, the Pro content did not change significantly. After 12 h of low temperature stress, the increase in content of Pro in the plant was saturated. The ROS content gradually increased as the time of stress was extended (Fig. 2e) with significant or highly significant differences between the treatment and the control groups. The activities of POD, SOD, and CAT were simultaneously significantly enhanced (Fig. 2f – h) and were effective at eliminating ROS and limiting their damage to plants.

In conclusion, low temperature stress significantly changed the morphology, physiological and cell solutes of *He. virescens*, damaging the tolerance and stability of leaf cells and suppressing their growth. Moreover, the low temperature produced some of the osmotic regulatory hormones to alleviate low temperature damage, but specific responses and regulatory mechanisms are still unclear. Therefore, additional research is merited.

### Sequence assembly and splicing

A high Illumina HiSeq™ flux sequencing platform was used to sequence the transcriptome of *He. virescens* leaves after 18 samples that been subjected to low temperature stress and obtained 24,000,000 bp raw data. After assembling and removing the redundancy, high-quality sequence data that was 22,730,000 bp long was obtained. Each sample of clean reads exceeded 6.5 G, indicating a high sequencing depth. The base error rate of the sequenced column for each sample was 0.02-0.03%, and Q20 and Q30 were > 98.0 and 94.0%, respectively, and the GC contents were > 50.21-55.21% (Table S1).

The clean reads had 396,649 transcriptional sequences obtained through Trinity splintering that were used as reference sequences for subsequent analysis. Table S2 shows the statistical results of reference alignment. A comparison of the efficiency between clean reads of 18 samples and the reference genome was higher. All were > 70%, indicating that they met the subsequent analytical needs. The transcriptome sequences were assembled into 112,775 Unigenes through Corset hierarchical clustering for a subsequent correlation analysis. The transcripts of N50 and N90 of the Unigene were 1579, 549, 1426, and 452, respectively. Larger N50 and N90 values showed the overlapped transcripts (Tables S3 and S4).

### Gene functional annotation

We annotated the unigenes obtained for gene functions using seven major databases (Nr, Nt, Pfam, KOG, Swiss-Prot, KO, and GO) (Table 1). There were 57,147 Unigenes (43.09%) in the Nt database, 60,901 Unigenes (45.92%) in the Nr database, 45,144 Unigenes (34.04%) in the Pfam database, 45,140 Unigenes (34.03%) in the GO database, and 39,512 Unigenes (29.79%) in the Swiss-Prot database. There were 18,765 Unigenes (14.15%) in the KO database and 10,100 Unigenes (7.61%) in the KOG database. It is worth noting that the NCBI nucleic acid sequence database (Nt) and NCBI protein sequence database (Nr) had higher annotation rates.

**Table 1:** Statistics of gene annotation

Database	Number of Unigenes	Percentage/%
Annotated in NR	60,901	45.92
Annotated in NT	57,147	43.09
Annotated in KO	18,765	14.15
Annotated in SwissProt	39,512	29.79
Annotated in PFAM	45,144	34.04
Annotated in GO	45,140	34.03
Annotated in KOG	10,100	7.61
Annotated in all Databases	7449	5.61
Annotated in at least one Database	132,612	100
Total Unigenes	132,612	100

**Fig. 3:**
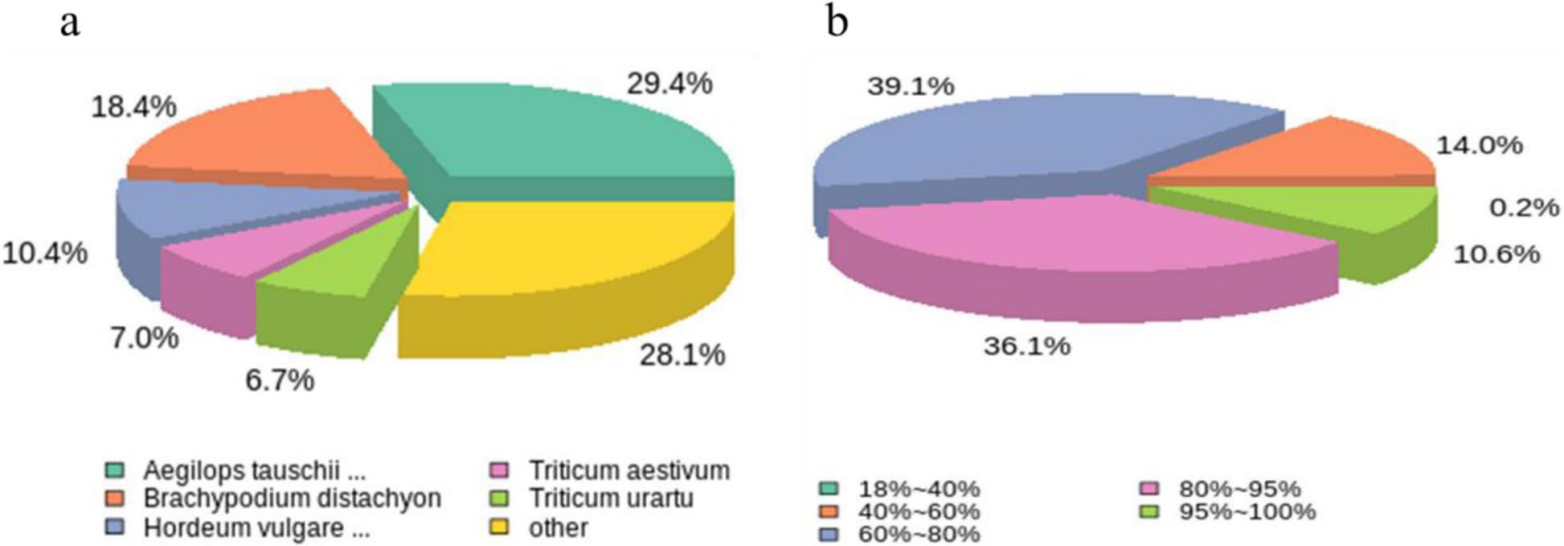
**A** Species distribution. **B** Sequence similarity distribution

By comparing the results of the comments with the NR library, the rate of homologous gene similarity was the highest with the *He. virescens* was the highest (29.4%), followed by *Brachypodium distachyon* (18.4%), barley (*Hordeum vulgare*) (10.4%), wheat (*Triticum aestivum)* (7.0%), *T. urartu* (6.7%) and other species (28.1%) (Fig. 3a). In conclusion, the highest rate of distribution of Unigene was in the NR database, indicating that genomes of the two species were the most similar. However, a few of them were in *B. distachyon*, *Ho. vulgare,* and *T. aestivum*, indicating the evolutionary overlap between the *He. virescens* and these species. In the sequence of *He. virescens*, 10.6% of the genes are 95-100% similar; 36.1% are 80-95% similar, 39.1% are 60-80% similar, and 14% are 40-60% similar (Fig. 3b).

### Analysis of differential gene expression

**Fig. 4:**
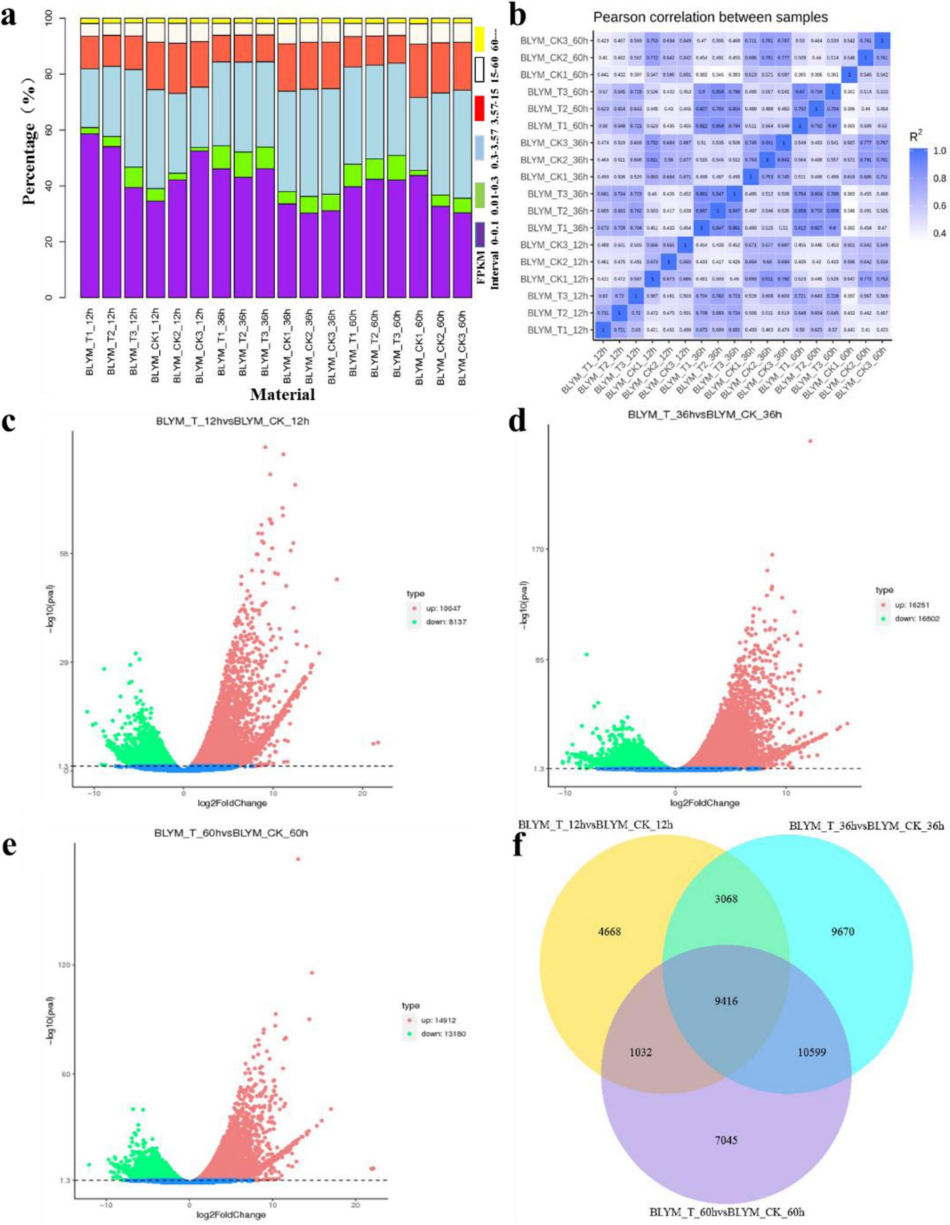
**a**: FPKM interval. Bar: The abscissa is the number distribution of genes in each FPKM interval. Different colors represent different expression intervals. In this figure, the overall distribution of FPKM of each sample can be compared and viewed. **b**: Heat map of the correlation coefficient between samples. Note: The abscissa is log10 of sample 1 (FPKM+ 1), the ordinate is log10 of sample 2 (FPKM+ 1), and R2 is the square of Pearson’s correlation coefficient. **c**, **d** and **e**: Volcano map of differential genes. Note: In the figure, the abscissa is Log2 Fold Change value, and the ordinate is -Log10 padj or -Log10 *p*-value. The dashed blue line represents the threshold line of differential gene screening criteria. **f**: Venn diagram of different genes after 12 h, 36 h and 60 h cryogenic treatment

Based on the expression profile, the level of gene expression was divided into six grades (Fig. 4a). The values of very low, low, moderate, high, and ultra-high and overexpressed levels of gene expression included 0 ~ 0.1, 0.1 ~ 0.3, 0.3 ~ 3.57, 3.57 ~ 15, 15 ~ 60, and > 60, respectively. By increasing the level of expression, its proportion gradually decreased. Compared with the control group, the proportion of extremely low, low, and ultra-high expressed genes was higher in the treatment group. In contrast, the balance of moderate, high, and overexpressed genes was less well distributed. A correlation analysis on the values of expression genes of all the samples was conducted using the FPKM (Fig. 4b). The patterns of expression within the group were highly similar, but differences between them were greater. DEGseq was used to screen the DEGs between the low temperature treatment and control groups (fold-change>2, FDR<0.01). The low 12 h treatment resulted in 18,184 differentially expressed genes (DEGs) (Fig. 4c). Among them, 10,047 were up-regulated, and 8137 were down-regulated. A total of 32,753 genes were differentially expressed after 36 h of low temperature treatment (Fig. 4d). Among them, 16,251 were up-regulated, and 16,502 were down-regulated. At 60 h of the low temperature treatment (Fig. 4e), 28,092 genes were differentially expressed, and 14,912 of them were up-regulated, while 13,180 were down-regulated. Thus, the levels of expression of the genes in *He. virescens* leaves changed significantly under low temperature stress.

### GO enrichment of the DEGs

To better explain the biological function of DEGs, a GO enrichment analysis was performed on them after 12 h, 36 h, and 60 h of treatment, which showed that 18,184 DEGs were enriched into 2675 biological processes. A total of 28 biological processes were significantly enriched after 12 h at low temperature. Ten cellular components significantly enriched a total of 1231 molecular functions with 27 of them significantly enriched. After 36 h of treatment, 32,753 DEGs were enriched in 2920 Biological processes with 62 of them significantly enriched. Among the 657 Cellular components, four were significantly enriched. Among 1394 enriched molecular functions, 15 were significantly enriched. After 60 h of treatment, 28,092 DEGs were enriched in 2846 biological processes, with 45 significantly enriched. During the process of enrichment of 645 Cellular components, nine were significantly enriched. Ten of the 1344 enriched molecular functions were significantly enriched.

The first 20 biological processes with significantly enriched genes were analyzed after 12 h, 36 h, and 60 h of treatment. Eleven Biological processes with DEGs were co-enriched and related to carbohydrate catabolism and metabolism (Fig. S1). Carbohydrate and sugar metabolism are part of respiration [17], which along with photosynthesis, are important for carbohydrate metabolism and sensitive to temperature [18]. The low temperature stress can reduce the accumulation of carbohydrates in plants and affect cell osmotic compounds or cryoprotectants. Therefore, it was hypothesized that photosynthesis and respiration determine carbohydrate metabolism, which affects the improvement of cold tolerance in plants.

In addition, the DEGs are enriched in specific biological processes, such as protein phosphorylation, protein denaturation, oxidative stress, peroxidase reactions, water response, and oxygen-containing reactions.

### KEGG enrichment of the DEGs

After 12 h, 36 h and 60 h of low temperature treatment, 9416 DEGs were enriched into 118 metabolic pathways (Table S5; Fig. 4f). Among them, 13 pathways were significantly enriched, and primarily included photosynthesis - antenna proteins (67 DEGs), circadian rhythm (35 DEGs), starch and sucrose metabolism (104 DEGs), plant-pathogen interaction (92 DEGs), galactose metabolism (39 DEGs), phenylpropanoid biosynthesis (86 DEGs), plant hormone signal transduction (71 DEGs), flavonoid biosynthesis (19 DEGs), photosynthesis (29 DEGs), alpha-linolenic acid metabolism (28 DEGs), amino sugar and nucleotide sugar metabolism (51 DEGs), carbon fixation in photosynthetic organisms (41 DEGs) and diterpenoid biosynthesis (11 DEGs).

**Fig. 5:**
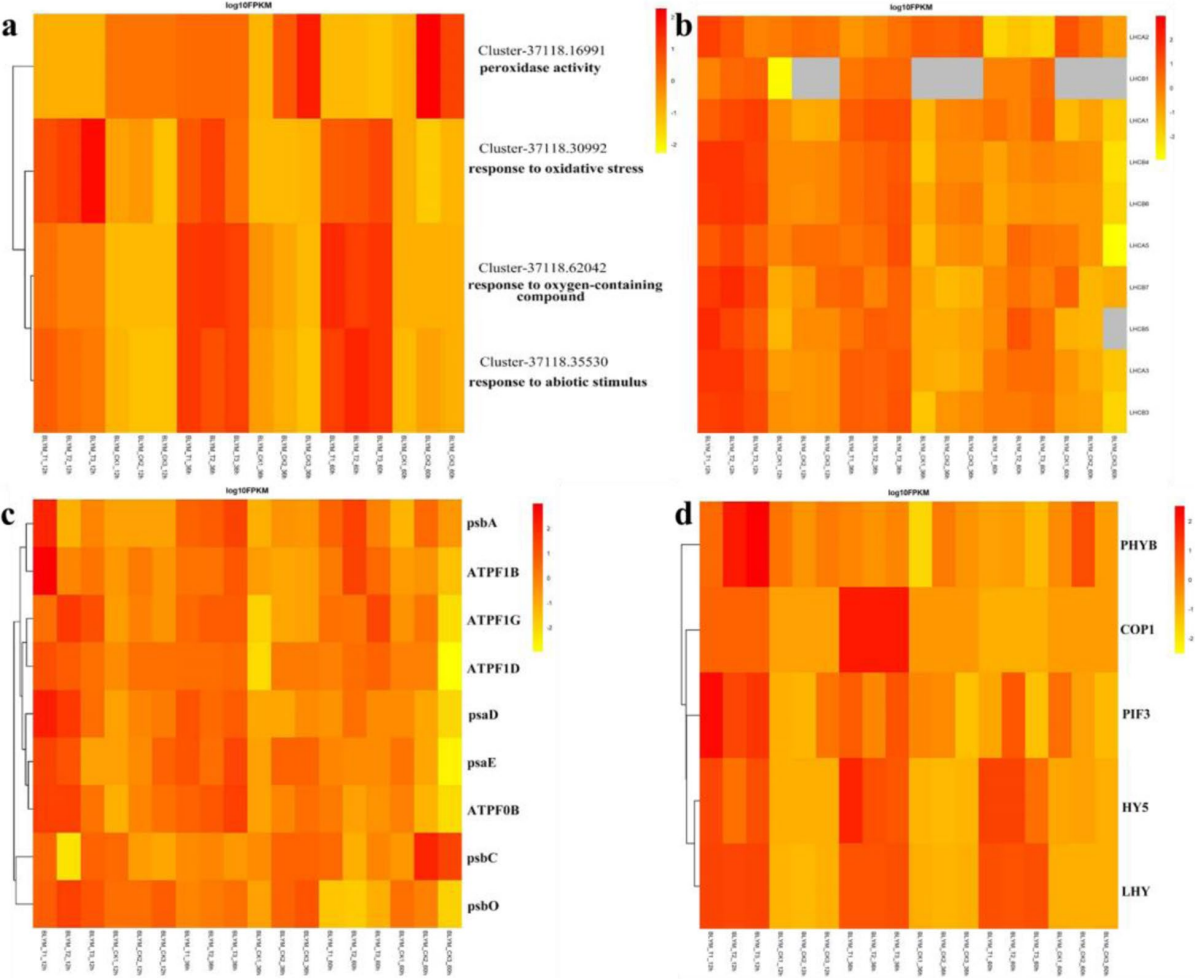
**a** Heat maps of differentially expressed genes in related biological processes. **b** Heat map of differentially expressed genes in the photosynthetic pathway. **c** Heat map of differentially expressed genes in the photosynthesis-antenna protein pathway. **d** Heat maps of differentially expressed genes in the plant-circadian pathway. BLYM: *Helictotrichon virescens*

A KEGG analysis of the DEGs at 12 h, 36 h, and 60 h of low temperature stress enriched 119, 120 and 120 metabolic pathways, respectively (Table S6-S8). Among them, 17, 15 and 11 pathways were significantly enriched, and nine pathways were co-enriched, including Photosynthesis – antenna proteins, Circadian rhythm – plant, Plant hormone signal transduction, Photosynthesis, Flavonoid biosynthesis, Pentose phosphate pathway, Phenylpropanoid biosynthesis, Fructose and mannose metabolism, and Plant-pathogen interaction (Fig. S2). In this study, three significantly enriched KEGG pathways, including Photosynthesis – antenna proteins, Circadian rhythm – plant and Photosynthesis, were analyzed in-depth. The results showed that 73, 74 and 70 DEGs annotated into the Photosynthesis – antenna proteins pathway at 12 h, 36 h and 60 h, respectively. Among them, *Lhca1*, *Lhca2*, *Lhca3*, *Lhca5*, *Lhcb1*, *Lhcb3*, *Lhcb4*, *Lhcb5*, *Lhcb6* and *Lhcb7* were up-regulated after low temperature treatment (Fig. 5b). Low temperature treatment up-regulated the expression of the genes that encode the PSI subunit (*psaA*, *psaD* and *psaE*) and PSII subunit (*psbA*, *psbC* and *psbO*) in the photosynthetic pathway (Fig. 5c). In addition, low temperature treatment up-regulated the genes that encode the ATPase subunits (*ATPF1B*, *ATPF1A*, *ATPF1G*, *ATPF1D* and *ATPF0B*) (Fig. 5c). In the Circadian rhythm – plant pathway, the DEGs encoded E3 ubiquitin-protein ligase (V), transcription factor HY5, MYB-associated transcription factor (LHY), and PHYB and PIF3, respectively. Among them, the levels of expression of COP1 and HY5 were up-regulated between the low temperature treatments (Fig. 5d). LHY is an MYB-related transcription factor and a morning gene that is part of the biological clock [14]. In this study, the initial level of expression level of LHY differed significantly between the control and low temperature treatment, and the low temperature treatment up-regulated its expression (Fig. 5d). The transcription factors that interact with photosensitive pigment B (PHYB) and photosensitive pigment (PIF3) are classified as “Morning genes” together with LHY, whose expression enhances the expression of PHYB and PIF3 in the biological clock. Thus, the levels of expression of these DEGs suggest that low temperatures themselves disrupt the circadian rhythm of *He. virescens*.

### Effects of low temperature stress on the expression of transcription factors in *Helictotrichon virescens*

**Fig. 6:**
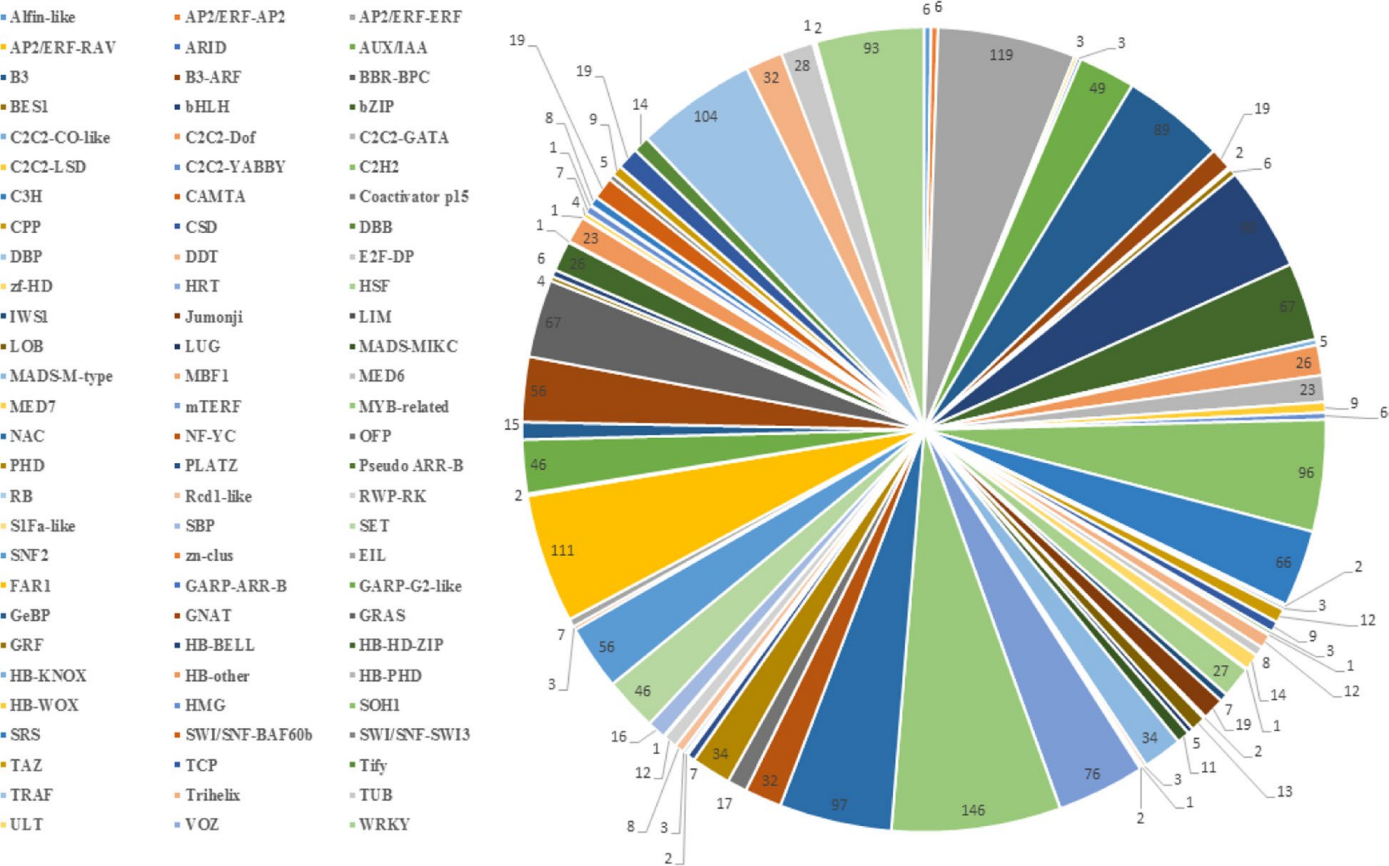
Distribution of differentially expressed transcription factors in different gene families

To investigate the effect of low temperature stress on the expression of transcription factors in *He. virescens*, low and normal temperature treatments were conducted, and the differential expression of transcription factor genes (TFs) was analyzed after transcriptome sequencing. The results showed that 2323 differentially expressed transcription factor genes were obtained under low temperature stress. All the TF genes obtained were classified into families, and all the TF genes of *He. virescens* were classified into 84 transcription factor families (Fig. 6). The top 10 transcription factor families were AP2/ERF, FAR1, TRAF, C2H2, bHLH, B3, bZIP/GRAS, C3H, AUX/IAA and GARP-G2-like.

**Fig. 7:**
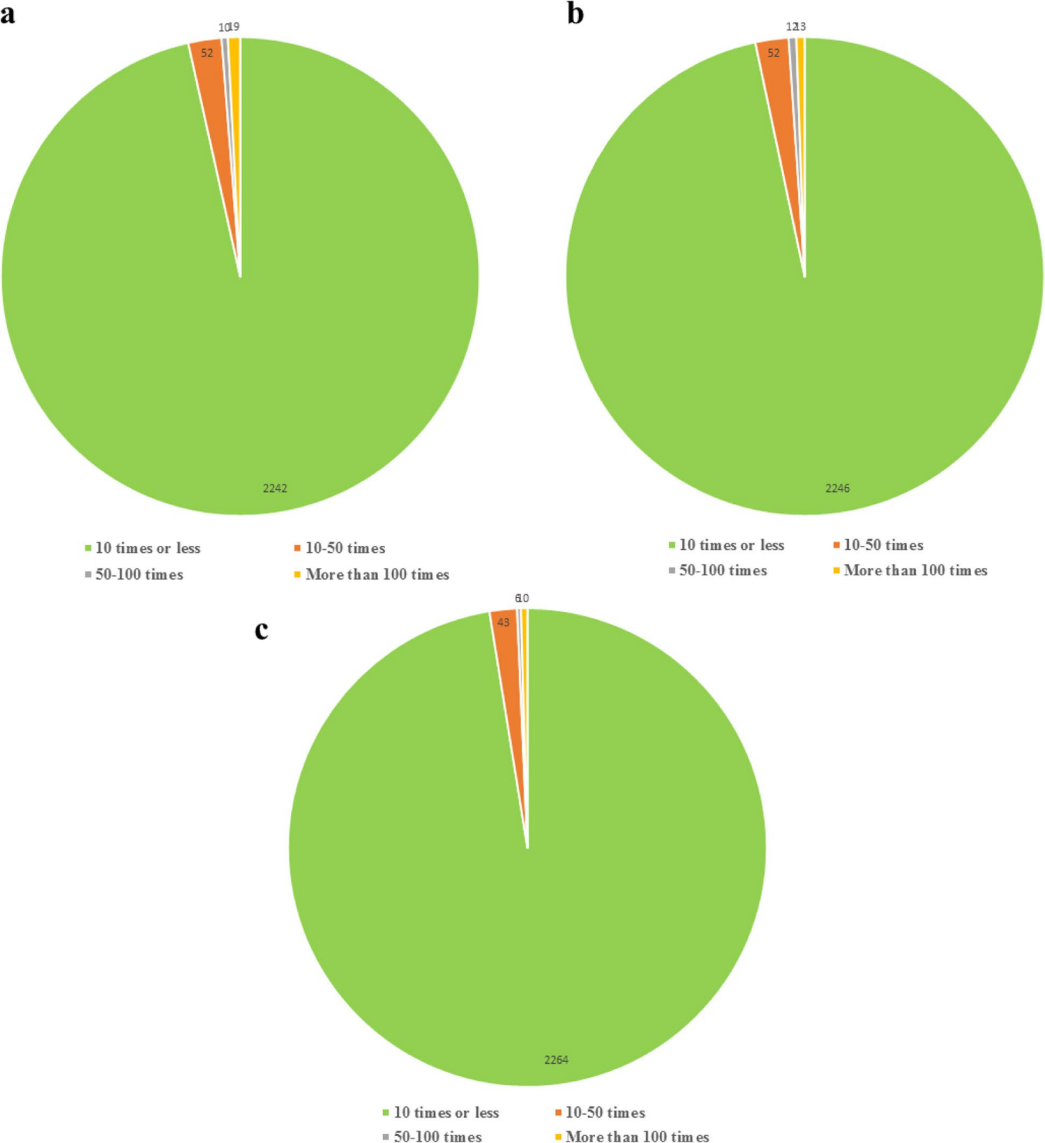
Fold change distribution of transcription factor genes Note: a, b and c are the expression multiples of transcription factor genes after 12 h, 36 h and 60 h low temperature treatment, respectively

The expression of multiples of TF genes was analyzed, and the TF genes that were differentially expressed under low temperature stress were classified by Ration (the ratio of FPKM value under low temperature stress to the FPKM value under normal temperature stress) as the classification index. The results showed that the distribution of multiple changes of transcription factor genes under different time treatments was basically the same (Fig. 7), with the most TF genes with an expression multiple < 10 times, the number of transcription factor genes contained after 12 h, 36 h and 60 h of treatment was 2242, 2246 and 2264, respectively. Among them, most of the TF genes (2264) had multiples < 10 times, and they had been subjected to low temperature for 60 h. After 12 h of low temperature treatment, the number of transcription factor genes with multiples > 100 times was the highest (19). After 36 h of low temperature treatment, most of the TF had multiples of expression between 50 and 100 times (12). Thus, most TF genes have few changes under low temperature stress and little influence on the regulation of gene expression. Only a few TF genes have undergone drastic changes, which has a substantial influence on the regulation of gene expression.

The TFs with large differential multiples were analyzed, and the TF genes with the top 15 differential expression multiples were selected for analysis under different treatment times. The results showed that all the transcription factors were up-regulated after 12 h of low temperature treatment (Tables 2, 3 and 4). Six of these transcription factors were members of the AP2/ERF family. Two were members of the C2H2 family, and the bZIP family had the highest rate of differential expression. After 36 h of low temperature treatment, all the TFs were up-regulated. Six of the transcription factors were members of the AP2/ERF family, three of the C2H2 family, and the bZIP family had the highest rate of differential expression. After 60 h of low temperature treatment, all the transcription factors were up-regulated. Five transcription factors were in the AP2/ERF family; two were from the C2H2 family, and two were from the MYB family. There are different types of TF families with large multiple differences between the treatments at different times, and most of these transcription factors are related to plant resistance, primarily concentrated in the AP2/ERF, MYB, bZIP and C2H2 families.

**Table 2:** Transcription factors with large difference multiple under low temperature stress (after 12 h treatment)

Gene ID	Transcription factor family	Fold change
Cluster-37,118.4686-2R	bZIP	88,341.3333
Cluster-37,118.77201-0R	Others	6598.2979
Cluster-37,118.4799-1R	C2H2	6392.1111
Cluster-37,118.77199-2R	Others	3195.1250
Cluster-37,118.77320-1R	AP2/ERF- > AP2/ERF-ERF	2227.8444
Cluster-37,118.11780-2F	AP2/ERF- > AP2/ERF-ERF	1249.2024
Cluster-37,118.77321-0R	AP2/ERF- > AP2/ERF-ERF	1183.3407
Cluster-37,118.72253-2F	MYB- > MYB-related	538.0743
Cluster-37,118.3964-1R	AP2/ERF- > AP2/ERF-ERF	306.3556
Cluster-37,118.4863-0R	C2H2	253.1799
Cluster-37,118.56181-2R	Others	247.4655
Cluster-37,118.28893-2R	Others	200.5954
Cluster-37,118.54292-1F	DBB	186.1898
Cluster-37,118.1583-2F	AP2/ERF- > AP2/ERF-ERF	182.6875
Cluster-37,118.77341-1F	AP2/ERF- > AP2/ERF-ERF	149.0179

**Table 3:** Transcription factors with large difference multiple under low temperature stress (after 36 h treatment)

Gene ID	Transcription factor family	Fold change
Cluster-37,118.4686-2R	bZIP	3500.3750
Cluster-37,118.4799-1R	C2H2	1093.4699
Cluster-37,118.3361-0F	bHLH	831.0000
Cluster-37,118.4866-1F	C2H2	829.4348
Cluster-37,118.77199-2R	Others	758.7011
Cluster-37,118.77201-0R	Others	678.4683
Cluster-37,118.4864-2F	C2H2	295.2523
Cluster-37,118.3966-0R	AP2/ERF- > AP2/ERF-ERF	189.6154
Cluster-37,118.72253-2F	MYB- > MYB-related	185.3746
Cluster-37,118.1583-2F	AP2/ERF- > AP2/ERF-ERF	123.5714
Cluster-37,118.77320-1R	AP2/ERF- > AP2/ERF-ERF	110.0485
Cluster-37,118.28893-2R	Others	104.2324
Cluster-37,118.3964-1R	AP2/ERF- > AP2/ERF-ERF	103.9017
Cluster-37,118.11780-2F	AP2/ERF- > AP2/ERF-ERF	99.9809
Cluster-37,118.77321-0R	AP2/ERF- > AP2/ERF-ERF	92.8544

**Table 4:** Transcription factors with large difference multiple under low temperature stress (after 60 h treatment)

Gene ID	Transcription factor family	Fold change
Cluster-37,118.77201-0R	Others	3350.0000
Cluster-37,118.77199-2R	Others	662.9641
Cluster-37,118.4686-2R	bZIP	385.6364
Cluster-37,118.4799-1R	C2H2	229.1000
Cluster-37,118.3964-1R	AP2/ERF- > AP2/ERF-ERF	161.6961
Cluster-37,118.41131-0R	Others	160.0000
Cluster-37,118.72253-2F	MYB- > MYB-related	154.8268
Cluster-37,118.3966-0R	AP2/ERF- > AP2/ERF-ERF	152.0417
Cluster-37,118.4550-1F	MYB- > MYB	152.0000
Cluster-37,118.77390-0R	C2H2	103.2381
Cluster-37,118.28893-2R	Others	98.7612
Cluster-37,118.78083-0F	AP2/ERF- > AP2/ERF-ERF	97.6000
Cluster-37,118.77321-0R	AP2/ERF- > AP2/ERF-ERF	87.8077
Cluster-2448.0-0R	AP2/ERF- > AP2/ERF-ERF	80.0000
Cluster-37,118.56181-2R	Others	57.3731

### RT-PCR verified the results of transcriptome sequencing

**Fig. 8:**
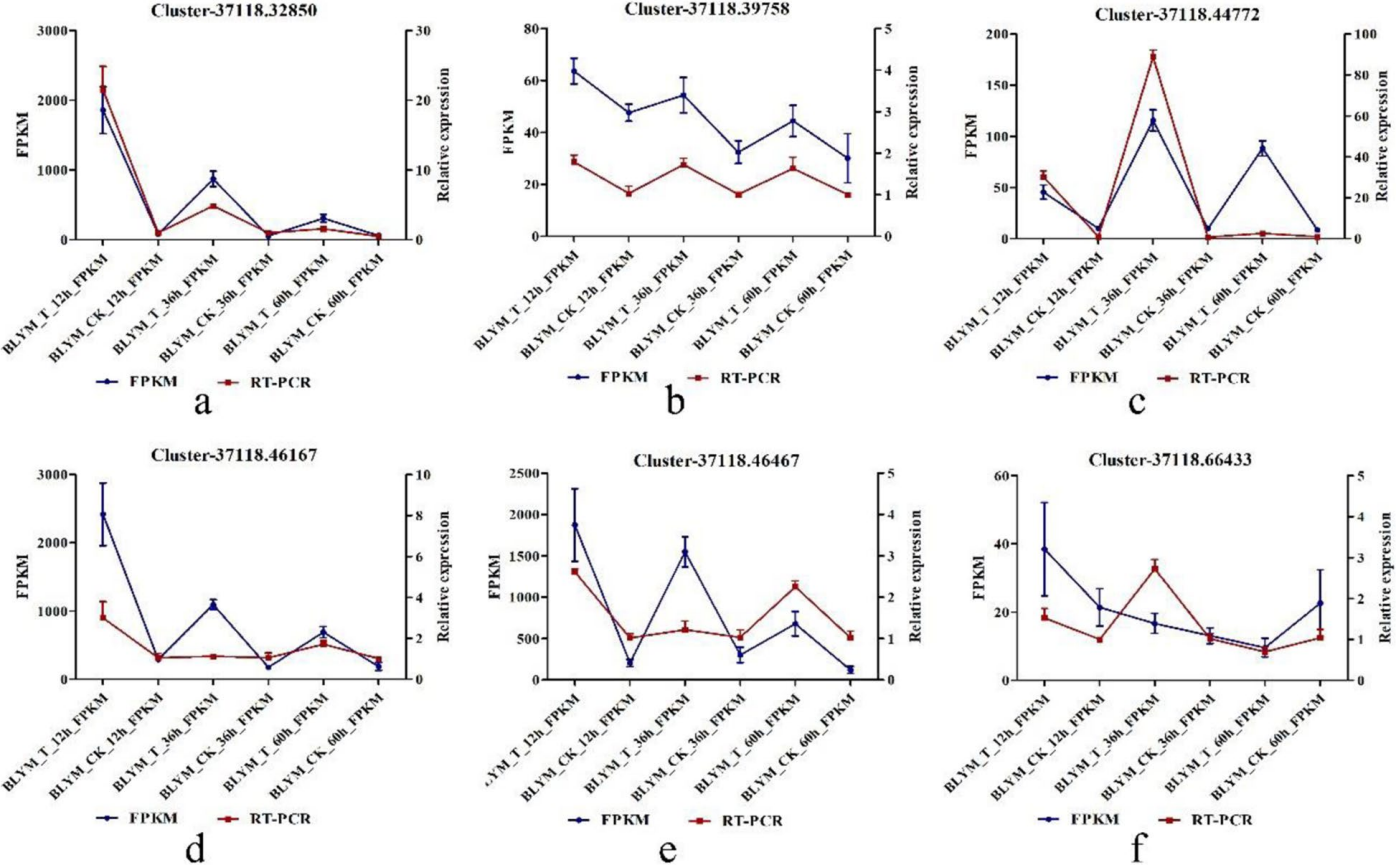
RNA-seq results verified by RT-PCR

To verify the accuracy of RNA-Seq, we randomly selected six DEGs involved in Circadian rhythm, Plant hormone signal transduction, Photosynthesis and Photosynthetic – antenna protein pathway for RT-PCR. The up-regulated or down-regulated DEGs in qPCR were consistent with the results from the RNA-Seq analysis (Fig. 8).

## Discussion

### *Helictotrichon virescens* provides an advantage for natural grassland improvement and ecological environment greening in the Qinghai-Tibet plateau area

An NR database comparison indicated that the species with the highest homologous gene similarity with *He. virescens* was *B distachyon*, followed by barley and wheat, and there was a partial overlap between the *He. virescens* and these species in evolution. *B. distachyon* is an annual herb that originated in western Asia and is highly adaptable. Barley and wheat are annual plants that are found at high elevations. During the course of evolution, the unique biological characteristics of Oatmeal have formed. Its perennial attributes and strong cold tolerance provide a guarantee for the improvement of natural grassland and the greening of ecological environment in the Qinghai-Tibet Plateau.

### *Helictotrichon virescens* reduces ROS damage by increasing the activity of antioxidant enzymes during cold stress

ROS are the natural byproduct of normal oxygen metabolism and play essential roles in cell signal transduction and homeostasis. Plants under adverse environmental conditions produce excessive amounts of ROS, which can damage cell structures and cause oxidative stress. Simultaneously, plants have ROS scavenging mechanisms, including enzymatic and non-enzymatic systems, in which antioxidant enzymes are important ROS scavengers. In this study, the ROS content gradually increased as the treatment time was extended (Fig. 2e). In particular, after 12 h and 36 h of low temperature stress, the ROS contents differed significantly between the treatment and control groups. After 60 h of low temperature stress, the ROS content differed significantly between the treatment and control groups.

Moreover, low temperature stress up-regulated the genes that regulate the oxidative stress response (Cluster37118.30992) (Fig. 5a). In addition, by extending the treatment time, the activities of antioxidant enzymes, such as POD, SOD and CAT, gradually increased. In particular, after 12 h of low temperature stress, the activities of POD, SOD and CAT differed significantly between the treatment and control groups. After 36 h and 60 h of low temperature stress, the activities of POD, SOD, and CAT contents differ significantly between the treatment and control groups. Simultaneously, under low temperature stress, the gene Cluster37118.16911 regulated the activity of POD, and the gene Cluster37118.62042 up-regulated the response to oxygen-containing compounds, such as SOD, which was consistent with the results of physiological experiments (Fig. 2f – h). These results indicate that POD, SOD and CAT were effective at clearing the ROS and reducing the amount of ROS damage during low temperature and cold stress.

In addition, autophagy is another important process in the life cycle, which is usually triggered by starvation and ROS and promoted by sucrose non-ferment-associated kinase 1 (SnRK1) or autophagy-associated (ATG) signaling [19]. Autophagy is a conservative cellular process that circulates cytoplasmic components. It is maintained at a basic level under normal conditions and is activated under stress conditions, such as carbon starvation, oxidative stress and sugar overload, in eukaryotes [19, 20]. Autophagy is crucial for nutrient cycling in plant vegetative organs [21]. Borja Belda-Palazon [22] showed that under normal growth conditions, SnRK2 and PP2C formed complexes with SnRK1, which inhibited the activity of SnRK1, enabling the growth promotion factor TOR to function normally and promote growth. Under adverse conditions, plants produce the hormone abscisic acid (ABA), which promotes the binding of PYR/PYL to PP2C and the cleavage of the SNRK2-PP2C-SNRK1 complex, which activates SnRK1 and inhibits TOR activity and growth. Since carotenoids are precursors to ABA, they can affect the content of this hormone [23]. In this study, low temperature stress inhibited the synthesis of carotenoids. As the treatment time was prolonged, the content of carotenoids gradually decreased, which further affected the content of ABA in plants.

### The content of chlorophyll a and b decreased during cold stress, which could be related to light protection

In this study, the leaf tissues of *He. virescens* attempted to reduce the absorption of light energy through optical antenna complexes (LHCs) during low temperature stress. The contents of chlorophyll a and b in the treatment and control groups did not differ significantly after 12 h of treatment with low temperature (Fig. 2a and b). After 36 h and 60 h of low temperature treatment, the content of chlorophyll a and b differed significantly between the treatment and control groups. In addition, the genes that encoded light-capture protein complexes, such as *Lhca1*, *Lhca2*, *Lhca3*, *Lhca5*, *Lhcb1*, *Lhcb3*, *Lhcb4*, *Lhcb5*, *Lhcb6* and *Lhcb7*, were up-regulated after cold treatment. Cold treatment also up-regulated the genes that encode the PSI subunit (*psaA*, *psaD* and *psaE*) and those that encode the PSII subunit (*psbA*, *psbC* and *psbO*) (Fig. 5-b). These results indicated that the leaves tried attempted to reduce light absorption by reducing the chlorophyll content during low-tlow temperature treatment. Therefore, it is proposed that photoinhibition is a temporary or persistent form of photoprotection, which maybe plays an important role in the low-tlow temperature stress tolerance in *Helictotrichon He. Virescensvirescens*.

### Circadian rhythm plays an important role during cold stress in *Helictotrichon virescens*

Studies have shown that the circadian clock is the gateway to induce genes that are related to cold stress. LHY is a morning gene of the circadian clock. In perennial woody plants, winter conditions (short photoperiod and low temperature) disrupt the expression of LHY in chestnut trees (*Castanea* spp.), leading to high expression in the winter [24]. In this study, the DEG that encodes LHY was strongly up-regulated during the cold treatment of *He. virescens* (Fig. 5-d). HY5 regulates many genes through transcription, making it the central hub of the transcription network [25]. It induces 10% of the cold-induced genes in *A. thaliana* as a positive regulator of cold domestication [13]. In this study, the DEGs that encoded HY5 were strongly upregulated during the cold treatment of *He. virescens* (Fig. 5d). Therefore, it is hypothesized that the circadian rhythm pathway is important to the low temperature response of *He. virescens*.

### Transcriptional regulation of transcription factors is a key factor in the response of *Helictotrichon virescens* to low temperature stress

The transcriptional regulation of TF is a key part of the plant response to low temperature stress. Currently, the TFs involved in the signaling responses to low temperature include P2/ERF, NAC, WRKY, MYB, bZIP and ZFPs [26]. Each TF basically contains four independent structural domains, namely a DNA binding domain, transcriptional functional domain, nuclear localization signal and oligomerization site [27]. The main function of TFs in the regulatory network of gene expression is determined by their transcriptional functional domains. The results of this study showed that all the TF genes of *He. virescens* could be classified into 84 TF families (Fig. 6). The top 10 transcription factor families were AP2/ERF, FAR1, TRAF, C2H2, bHLH, B3, bZIP/GRAS, C3H, AUX/IAA and GARP-G2-like, and most of the transcription factor genes belong to the transcription factor family related to resistance. According to the analytical results of multiple differences of TF genes in this study (Fig. 7), transcription factor families with greater differential expression also contain transcription factor families related to resistance, such as AP2-ERF and bZIP, under different time treatments. The existence of these same types of TF genes related to resistance suggests that there are some common response mechanisms in *He. virescens* under low temperature stress.

Plant cold resistance is a quantitative trait controlled by multiple genes, and there are substantial differences among varieties with different cold resistance, and the types and levels of expression of TFs differentially expressed under low temperature stress differ. Changes in the levels of expression of specific TFs can substantially affect the ability of plants to adapt to adversity [28]. This study showed that, although the types and patterns of expression of TFs related to the cold tolerance of *He. virescens* at different time treatments generally had the same trend, there were still some differences, and the TFs under different time treatments showed some time specificity in their transcription levels. In terms of the levels of expression of TFs (Tables 2, 3 and 4), the TFs with large differences were primarily distributed in the bZIP, C2H2 and AP2/ERF families after 12 h of low temperature treatment. After 36 h of low temperature treatment, the TFs with large multiple differences were primarily distributed in the bZIP, C2H2 and bHLH families, while after 60 h of low temperature treatment, the TFs with large multiple differences were primarily distributed in bZIP, C2H2 and some unknown gene families. This unique TF family and unique transfactor genes with large multiple differences are likely to be an important reason for the enhancement of cold tolerance in *He. virescens* under different time treatments, and further study to clarify the transcriptional regulation mechanism of *He. virescens* under low temperature stress is merited.

## Conclusions

To elucidate the mechanism of response of *He. virescens* to cold stress, this study was based on a combination of physiological experiments and transcriptomic sequencing. At low temperature, the accumulation of ROS was the same as the activities of POD, SOD and CAT. The gene that regulates the activity of POD (Cluster37118.16911) and the gene that regulates ROS (Cluster37118.62042) could be involved in the elimination of ROS during low temperature treatment. During the low temperature stress period, the content of chlorophyll a and b decreased more and more with the delay of the treatment time. Among them, the difference between the control was not significant, and the difference between the control and the treatment was significant. At the same time, the expression of related differential genes was up-regulated during low temperature treatment. In other words, Decrease of content of chlorophylls (which can be also accompanied with decrease of total quantity of reaction centers) leads to an increase in photosynthetic damage. Finally, circadian pathways are essential in the response to cold stress in *He. virescens*. Among them, the DEG of LHY was strongly up-regulated under cold stress. The DEGs that encode HY5 were strongly up-regulated under cold stress.

This study provides an opportunity to fully understand the mechanism of response to cold stress of *He. virescens*. It raises questions in perennial herbaceous cold stress research, such as how leaves integrate light and low temperature signals to regulate the circadian rhythms of crops and how leaf tissue reduces light absorption and enhances light protection through the optical antenna complex (LHCs).

## Materials and methods

### Plant materials and growth conditions

**Fig. 9:**
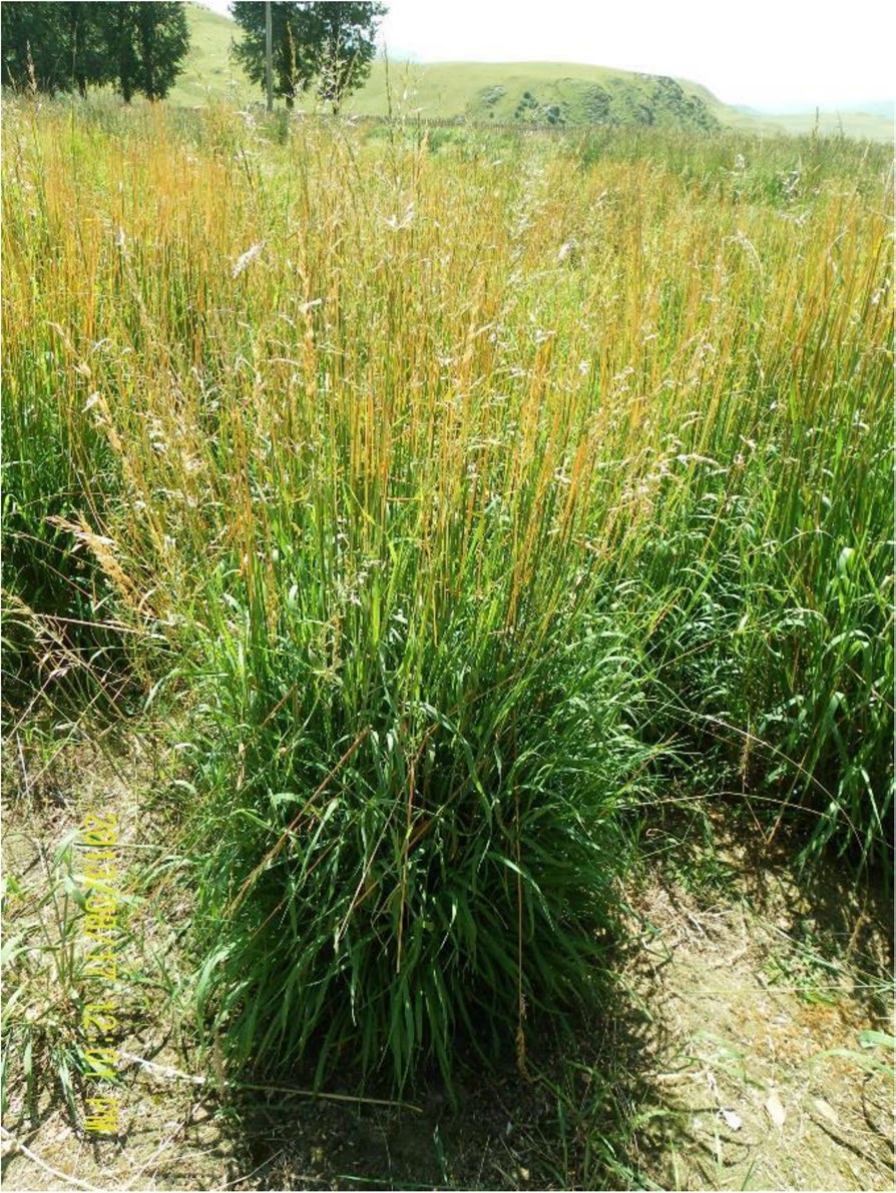
Photographs of Helictotrichon virescens

The Sichuan Grassland Work Station, the Grassland Station of Ganzi Prefecture, Sichuan Province and Sichuan Jinzong Liaoyuan Seed Industry Technology Co. Ltd. obtained a few seeds of *He. virescens* from the Ganzi Prefecture forage germplasm resource collection of Sichuan Province in 1999. The plants turned green early, showed better growth and forage quality during propagation (Fig. 9). After several years of selection and domestication, new varieties were bred, and in 2015, the Sixth China Grass Variety Approval Committee approved it as a wild cultivar. The cultivar applicants were Guangwu He, Ruizheng Zhang, Tao Ma, Dengkai Liu and Mingjiu Yao. The cultivar examination unit is the China Grass Variety Examination Committee, and the cultivar registration number was 493. *He. virescens* is the first and only forage of *Helictotrichon* approved by the state in China. Currently, the seeds are stored in a cryogenic freezer at the Sichuan Grass Industry Technology Research and Promotion Center (Sichuan, China).

This study used *H. virescens* as the experimental material. Seeds were sown in flowerpots (21 cm × 16 cm) that contained substrate (peat: pine needles: yellow clay [3:1:1] [v/v]. When the first leaves expanded fully, we selected eight strains of each material with three replications and transferred them into 1/2 Hoagland nutrient solution. Five-week-old samples were collected, immediately frozen in liquid nitrogen on the sampling day and stored at − 80 °C.

### Low temperature and cold stress treatment on *Helictotrichon virescens* plants

After the five-week-old seedlings were separated into control and treatment groups, they were treated with low temperatures stress at a constant temperature and light in an incubator (MODEL: MLR-352H-PC) of the Cell Genetics Laboratory of Maize Research Institute of Sichuan Agricultural University (Chengdu, China) utilizing a complete block design. The low temperature and control groups were constantly maintained at 0 °C and 25 °C, respectively, at an illumination of 3000 Lux. Subsequently, we obtained leaves after 12 h, 36 h and 60 h of low temperature treatment for RNA-Seq and related indices.

### Relative conductivity (REC)

The relative conductivity was measured as described by Xu [29]. A 2 × 4 cm leaf was removed from the middle of the first fully expanded leaf of each seedling. The leaves were mixed, cut into 1 cm segments, divided into three portions, transferred into 10 mL EP tubes, and finally the tubes were filled with distilled water. After the leaf blades had been soaked in water for 3 h, the EC1 was determined using a conductivity meter. The samples were then boiled for 10 min and cooled to room temperature. The EC2 was measured using a conductivity meter.

1 $$\mathrm{Relative}\ \mathrm{conductivity}:\mathrm{REC}=\mathrm{EC}1/\mathrm{EC}2\times 100\%.$$

### Determination of chlorophyll a and b and carotenoid contents

The chloroplast pigments were extracted by homogenizing 0.2 g of fresh leaves with 95% ethanol and centrifuging them for 10 min at 10,000 g at room temperature. The extracted chloroplast pigments were poured into a colorimetric cup that was 1 cm in diameter. The absorbance was measured at 663 nm, 646 nm and 470 nm in a UV-visible spectrophotometer (model UV-1800) using 95% ethanol as a blank [30]. The equations for calculations are as follows:

2 $$\mathrm{The}\ \mathrm{concentration}\ \mathrm{of}\ \mathrm{chlorophyll}\ \mathrm{a}=12.21\times \mathrm{OD}663-2.81\times \mathrm{OD}646$$

3 $$\mathrm{The}\ \mathrm{concentration}\ \mathrm{of}\ \mathrm{chlorophyll}\ \mathrm{b}=20.13\times \mathrm{OD}646-5.03\times \mathrm{OD}663$$

4 $$\mathrm{Concentration}\ \mathrm{of}\ \mathrm{carotenoids}=\left(1000\mathrm{A}470-3.27\ \mathrm{Cha}-104\mathrm{Chb}\right)/229$$

### Determination of ROS content and antioxidant enzyme activity

Test kits purchased from Suzhou Keming Biotechnology Co., Ltd. (Suzhou, China) were used to determine the contents of proline (Pro) and ROS and the activities of superoxide dismutase (SOD), peroxidase (POD) and catalase (CAT) using a UV-visible visible spectrophotometer following the manufacturer’s instructions.

### RNA extraction and detection

The Beijing Novogene Technology Co., Ltd. (Beijing, China) extracted the RNA and detected it.

### cDNA library construction and sequencing

The Beijing Novogene Technology Co., Ltd., constructed the cDNA library and sequenced it utilizing Oligo (dT) magnetic beads enriched mRNA with polyA tail procedures. Briefly, mRNA was randomly fragmented by divalent cations in NEB Fragmentation Buffer (NEB, Ipswich, MA, USA). The first strand of cDNA was prepared using random oligonucleotides as a primer, and the second strand was synthesized using DNA polymerase I. The ends of purified double-stranded cDNA were repaired by adding a tail and sequencing connector and screened to 250-300 bp cDNA for PCR amplification. The PCR product was purified again to obtain the final library, and after qualifying the library, an Illumina HiSeq™ 4000 sequencing platform (Illumina, Inc., San Diego, CA, USA) was used for sequencing.

### Data filtering and splicing assembly

High-quality sequence data was obtained by filtering the raw reads to a small number of reads that contained sequencing adaptors, and those that did not show the base information were considered low-sequencing reads. Statistical Q20 (Phred = −10log10 [e]), Q30 (Phred = −10log10 [e)], GC content and sequencing error (< 6%), and other indicators of sequencing quality control were used for these procedures.

Trinity software [31] was used to obtain the clean reads of transcripts for subsequent analysis. TGICL software was used to obtain Unigenes by homologous clustering for subsequent analysis. The splicing transcript was sequenced based on its length from long to short, and the length of transcript was added to the length of splicing transcript, so that it was not < 50%/90% of the total length, namely N50/N90, to measure the continuity of de novo assembly. Its numerical value can be used to evaluate the quality of the assembly.

### Unigenes function comment

BLAST was used to obtain Unigenes [32] and perform functional annotations in the following seven databases: NR, NT, gene ontology (GO), Eukaryotic Orthologous Groups (KOG), KO (Kyoto Encyclopedia of Genes and Genomes, KEGG), Swiss-Prot, and fragments per kilobase of transcript per million mapped fragments (FPKM).

### Screening and functional annotation analysis of differentially expressed genes

The RSEM method [33] was to quantitatively analyze gene expression. The clean data of all the samples were compared to the reference sequence obtained by Trinity splicing, and the read count to a gene was obtained using the Bowtie2 method. The read count was converted to an FPKM, and the level of gene expression level was evaluated using the FPKM value [29, 34] (FPKM > 0.3 was regarded as gene expression).

The level of differentially expressed genes (DEGs) was screened between different samples by comparing DEGseq software [35] to the original read count for standardization through the negative binomial distribution hypothesis test probability statistical model (*P*-value) calculation and finally (BH) adjustment for multiple hypothesis testing using the FDR value (False Discovery Rate, namely padj), |log_2_(FoldChange)| > 1 and padj < 0.05 was considered as the standard for differential gene screening.

A GO biological enrichment analysis was performed on the differential gene set using goseq [36], and KEGG metabolism and signal transduction pathway enrichment analyses were performed on the differential gene set using KOBAS [37]. padj < 0.05 was used as the threshold of significant enrichment in both of the analyses described above.

### Real-time PCR validated the transcriptome results

To verify the accuracy of transcriptome sequencing results, six genes were randomly selected for real-time PCR detection. Primer5.0 was used to design differential gene sequence primers (Table S9) with *Actin* as the internal reference gene. The 2^-ΔΔ^CT method was used to calculate the relative level of gene expression, and three biological replicates and three technical replicates were established for each sample. PCR amplification was performed in a 10 μL reaction volume that contained 1 μL cDNA, 5 μL qPCR Super mix, 0.4 μL forward/reverse primer, and 3.2 μL nuclease-free water. The reaction conditions were established as pre-denaturation 95 °C, 3 min; denaturation 95 °C, 10 s; annealing at 60 °C, 30 s; extension 72 °C, 30 s, and 40 cycles.

## Supplementary Information

**Additional file 1: Figure S1**. The 20 most enriched biological process GO terms among DEGs. **Figure S2**. The 20 most enriched KEGG pathway among DEGs. **Table S1**. Summary of the quality of sample sequencing data. **Table S2**. Sample comparison statistics. **Table S3**. Frequency distribution of splicing length. **Table S4**. Splicing length distribution. **Table S5**. The number of co-enriched KEGG pathways and differentially expressed genes. **Table S6**. KEGG pathway enriched by DEGs after 12 h of cryogenic treatment. **Table S7**. KEGG pathway enriched by DEGs after 36 h of cryogenic treatment. **Table S8**. KEGG pathway enriched by DEGs after 60 h of cryogenic treatment. **Table S9**. Real time PCR genes and their primers.

## Data Availability

The datasets supporting the conclusions of this article are included within the article and its additional files. The RNA-seq raw data have been submitted to the Short Read Archive (SRA) data library under accession number PRJNA810780. The address is as follows: https://www.ncbi.nlm.nih.gov/sar/PRJNA810780.
