# Supplementary material for: Physiological attributes and transcriptomics analyses reveal the mechanism response of Helictotrichon virescens to low temperature stress
Source: BMC Genomics. 2022 Apr 7;23:280. doi: 10.1186/s12864-022-08526-4 (PMC8991566; doi:10.1186/s12864-022-08526-4)
Supplement: Supplementary file 1 — Additional file 1: Figure S1. The 20 most enriched biological process GO terms among DEGs. Figure S2. The 20 most enriched KEGG pathway among DEGs. Table S1. Summary of the quality of sample sequencing data. Table S2. Sample comparison statistics. Table S3. Frequency distribution of splicing length. Table S4. Splicing length distribution. Table S5. The number of co-enriched KEGG pathways and differentially expressed genes. Table S6. KEGG pathway enriched by DEGs after 12 h of cryogenic treatment. Table S7. KEGG pathway enriched by DEGs after 36 h of cryogenic treatment. Table S8. KEGG pathway enriched by DEGs after 60 h of cryogenic treatment. Table S9. Real time PCR genes and their primers. [file 12864_2022_8526_MOESM1_ESM.pdf]

Additional file 1: Supplementary Figs. S1 to S2. **Fig S1.** The 20 most enriched biological process GO terms among DEGs. **Fig S2.** The 20 most enriched KEGG pathway among DEGs.

Additional file 2: Table S1. Summary of the quality of sample sequencing data. **Table S2.** Sample comparison statistics. **Table S3.** Frequency distribution of splicing length. **Table S4.** Splicing length distribution. **Table S5.** The number of co-enriched KEGG pathways and differentially expressed genes. **Table S6.** KEGG pathway enriched by DEGs after 12h of cryogenic treatment. **Table S7.** KEGG pathway enriched by DEGs after 36h of cryogenic treatment. **Table S8.** KEGG pathway enriched by DEGs after 60h of cryogenic treatment. **Table S9.** Real time PCR genes and their primers.

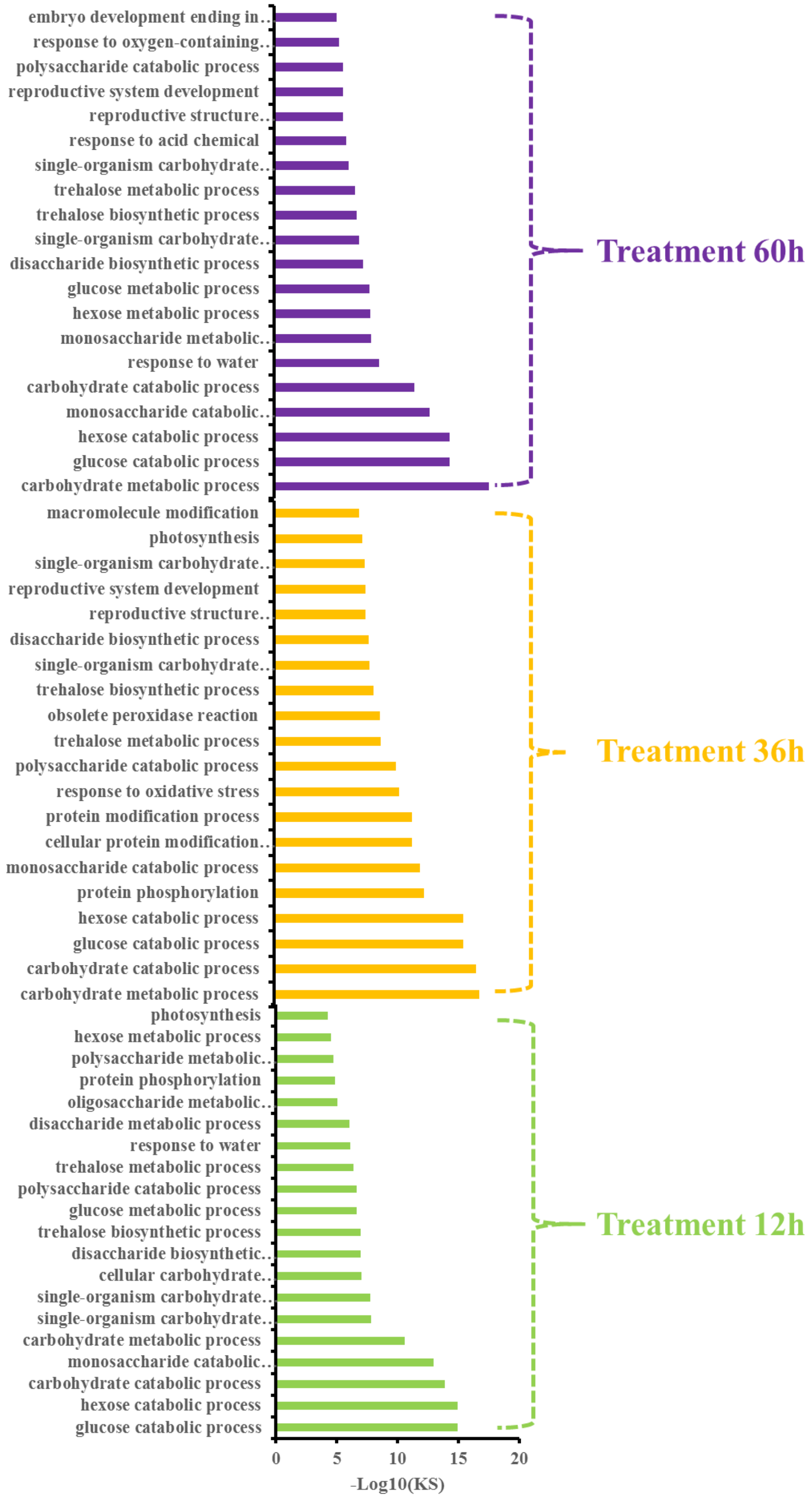

Fig. S1. The 20 most enriched biological process GO terms among DEGs.

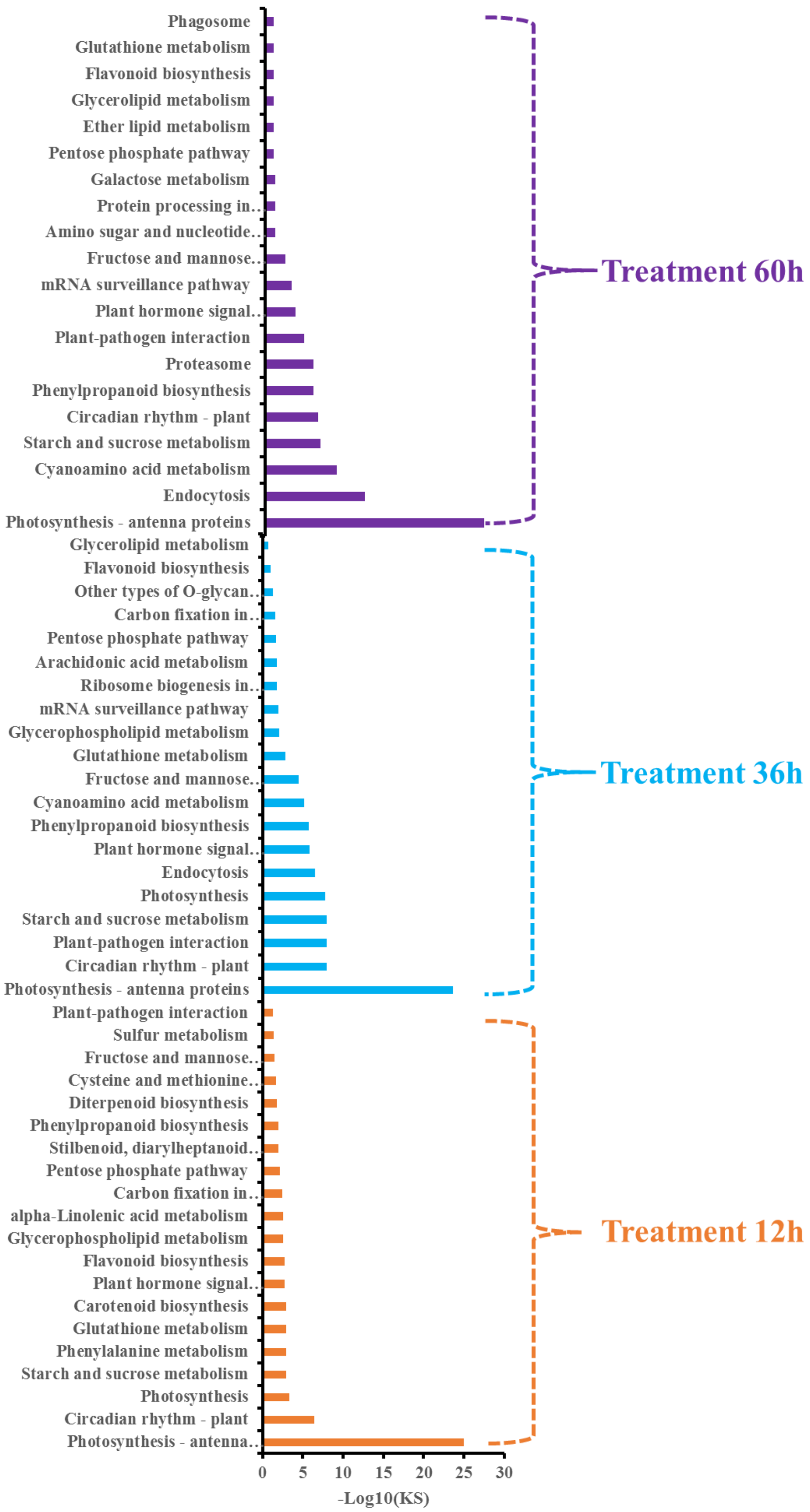

Fig. S2. The 20 most enriched KEGG pathway among DEGs.

Table S1. Summary of the quality of sample sequencing data

Sample	Raw Reads	Clean Reads	Clean Bases	Error (%)	Q20 (%)	Q30 (%)	GC Content (%)
BLYM_T1_12h	26220673	24464993	7.34G	0.02	98.2	94.72	52.33
BLYM_T2_12h	24004815	22738309	6.82G	0.02	98.33	94.88	54.06
BLYM_T3_12h	23474948	22151213	6.65G	0.02	98.36	94.96	53.96
BLYM_CK1_12h	22726162	21897098	6.57G	0.02	98.22	94.56	51.24
BLYM_CK2_12h	23249369	22633324	6.79G	0.02	98.21	94.52	50.81
BLYM_CK3_12h	22787961	21781005	6.53G	0.02	98.25	94.65	50.89
BLYM_T1_36h	22962757	22131529	6.64G	0.02	98.2	94.62	54.95
BLYM_T2_36h	22752893	21688555	6.51G	0.02	98.29	94.85	55.19
BLYM_T3_36h	22769177	21858525	6.56G	0.02	98.21	94.67	55.21
BLYM_CK1_36h	25642325	24347509	7.3G	0.02	98.25	94.64	51.96
BLYM_CK2_36h	23168551	22200273	6.66G	0.02	98.1	94.3	52.84
BLYM_CK3_36h	23471548	22426001	6.73G	0.02	98.24	94.71	52.75
BLYM_T1_60h	22820515	21930815	6.58G	0.02	98.21	94.63	54.39
BLYM_T2_60h	23824143	22046791	6.61G	0.02	98.23	94.62	53.06
BLYM_T3_60h	23171958	21861459	6.56G	0.02	98.3	94.84	53.97
BLYM_CK1_60h	23350518	22600629	6.78G	0.02	98.13	94.3	50.21
BLYM_CK2_60h	24005475	23344195	7G	0.02	98.32	94.77	51.34
BLYM_CK3_60h	26178364	25077674	7.52G	0.02	98.36	94.84	50.87

Notes： BLYM, Helictotrichon *virescens*

Table S2. Sample comparison statistics

Sample name	Total reads	Total mapped
BLYM_T1_12h	48929986	34228598(69.95%)
BLYM_T2_12h	45476618	32872076(72.28%)
BLYM_T3_12h	44302426	32847264(74.14%)
BLYM_CK1_12h	43794196	31872530(72.78%)
BLYM_CK2_12h	45266648	31845926(70.35%)
BLYM_CK3_12h	43562010	30562470(70.16%)
BLYM_T1_36h	44263058	30862274(69.72%)
BLYM_T2_36h	43377110	31572844(72.79%)
BLYM_T3_36h	43717050	30723956(70.28%)
BLYM_CK1_36h	48695018	34889992(71.65%)
BLYM_CK2_36h	44400546	32472412(73.14%)
BLYM_CK3_36h	44852002	32785652(73.10%)
BLYM_T1_60h	43861630	31427724(71.65%)
BLYM_T2_60h	44093582	31692848(71.88%)
BLYM_T3_60h	43722918	32140950(73.51%)
BLYM_CK1_60h	45201258	31280868(69.20%)
BLYM_CK2_60h	46688390	33198830(71.11%)
BLYM_CK3_60h	50155348	36654242(73.08%)

Table S3. Frequency distribution of splicing length

Length_interval	300bp-500bp	500bp-1kbp	1kb-2kbp	>2kbp	Total
Number of transcripts	118241	137154	100489	57070	412954
Number of Unigenes	51785	41601	24908	14318	132612

Note: Length_interval: Indicates the different length intervals of spliced Transcript/Unigene; Number of transcripts: Transcript number in corresponding length interval; Number of Unigenes: Denotes the number of Unigene in the corresponding length interval

Table S4. Splicing length distribution

	Min_length	Mean_length	Median_length	Max_length	N50	N90	Total_nucleotides
Transcripts	301	1109	765	16758	1548	499	458118854
Genes	301	964	612	16758	1364	419	127830487

Note: Min_length: Transcript/ minimum length of Unigene. Mean_length: Transcript/ Average length of Unigene; Median_length: Transcript/Unigene median length; Max_length: Transcript/Unigene Max length. Total_nucleotides: Transcript/ total nucleotide count of Unigene.

Table S5. The number of co-enriched KEGG pathways and differentially expressed genes

Term	ID	DEGs number	P-Value
Photosynthesis - antenna proteins	ko00196	67	1.07E-25
Circadian rhythm - plant	ko04712	35	1.16E-08
Starch and sucrose metabolism	ko00500	104	2.87E-05
Plant-pathogen interaction	ko04626	92	4.66E-05
Galactose metabolism	ko00052	39	0.000311355
Phenylpropanoid biosynthesis	ko00940	86	0.000518136
Plant hormone signal transduction	ko04075	71	0.001084813
Flavonoid biosynthesis	ko00941	19	0.00171208
Photosynthesis	ko00195	29	0.002994406
alpha-Linolenic acid metabolism	ko00592	28	0.003944021
Amino sugar and nucleotide sugar metabolism	ko00520	51	0.007834603
Carbon fixation in photosynthetic organisms	ko00710	41	0.00829015
Diterpenoid biosynthesis	ko00904	11	0.008484416
Pentose phosphate pathway	ko00030	29	0.012546615
Glutathione metabolism	ko00480	43	0.017719574
Glycerophospholipid metabolism	ko00564	40	0.019382882
Arachidonic acid metabolism	ko00590	12	0.020992627
Cysteine and methionine metabolism	ko00270	48	0.028346534
Carotenoid biosynthesis	ko00906	18	0.031007261
Glycerolipid metabolism	ko00561	32	0.041955392
Regulation of autophagy	ko04140	15	0.044698067
Stilbenoid, diarylheptanoid and gingerol biosynthesis	ko00945	16	0.057454199
Glycolysis / Gluconeogenesis	ko00010	57	0.070722669
Phenylalanine metabolism	ko00360	21	0.076849454
Ether lipid metabolism	ko00565	12	0.085546554
Cyanoamino acid metabolism	ko00460	36	0.092145776

Fructose and mannose metabolism	ko00051	25	0.114297244
Monoterpenoid biosynthesis	ko00902	6	0.14692838
Riboflavin metabolism	ko00740	6	0.14692838
Steroid biosynthesis	ko00100	13	0.150948775
Sulfur metabolism	ko00920	17	0.169129435
Pentose and glucuronate interconversions	ko00040	18	0.170151119
Glycosphingolipid biosynthesis - globo series	ko00603	7	0.175934707
Ascorbate and aldarate metabolism	ko00053	16	0.17697477
Selenocompound metabolism	ko00450	12	0.178932583
Glycosaminoglycan degradation	ko00531	8	0.247140179
Monobactam biosynthesis	ko00261	5	0.247837292
Arginine and proline metabolism	ko00330	20	0.260985605
Other glycan degradation	ko00511	9	0.280799282
Endocytosis	ko04144	45	0.286318535
Nitrogen metabolism	ko00910	16	0.292838064
mRNA surveillance pathway	ko03015	32	0.294053321
Other types of O-glycan biosynthesis	ko00514	2	0.315313472
Terpenoid backbone biosynthesis	ko00900	18	0.319534472
Glycosphingolipid biosynthesis - ganglio series	ko00604	4	0.392887936
Lysine biosynthesis	ko00300	6	0.401443844
C5-Branched dibasic acid metabolism	ko00660	3	0.43028042
Taurine and hypotaurine metabolism	ko00430	5	0.456517728
Cutin, suberine and wax biosynthesis	ko00073	8	0.45777062
Fatty acid biosynthesis	ko00061	13	0.465224549
Ribosome biogenesis in eukaryotes	ko03008	23	0.491245254
Linoleic acid metabolism	ko00591	8	0.51044745
beta-Alanine metabolism	ko00410	13	0.519560273
Biosynthesis of unsaturated fatty acids	ko01040	10	0.559940949
Pantothenate and CoA biosynthesis	ko00770	9	0.56050899

Zeatin biosynthesis	ko00908	5	0.587551798
Thiamine metabolism	ko00730	4	0.61608111
Inositol phosphate metabolism	ko00562	16	0.616687712
Biotin metabolism	ko00780	5	0.627225751
Sphingolipid metabolism	ko00600	8	0.640524262
Tryptophan metabolism	ko00380	13	0.658103761
Brassinosteroid biosynthesis	ko00905	2	0.678772014
Protein export	ko03060	15	0.683844739
Indole alkaloid biosynthesis	ko00901	1	0.733407872
Tyrosine metabolism	ko00350	10	0.77414556
Isoquinoline alkaloid biosynthesis	ko00950	4	0.780238802
Glyoxylate and dicarboxylate metabolism	ko00630	31	0.783521121
Caffeine metabolism	ko00232	1	0.814135469
Butanoate metabolism	ko00650	7	0.815205362
Ubiquinone and other terpenoid-quinone biosynthesis	ko00130	10	0.82037717
Base excision repair	ko03410	8	0.822558742
Vitamin B6 metabolism	ko00750	2	0.855975857
Sulfur relay system	ko04122	2	0.880625921
Basal transcription factors	ko03022	10	0.8963918
Porphyrin and chlorophyll metabolism	ko00860	12	0.90215047
Folate biosynthesis	ko00790	3	0.905830504
Nicotinate and nicotinamide metabolism	ko00760	7	0.908135062
Phagosome	ko04145	14	0.908152122
Sesquiterpenoid and triterpenoid biosynthesis	ko00909	2	0.910366143
Glycosylphosphatidylinositol(GPI)-anchor biosynthesis	ko00563	4	0.913522202
RNA degradation	ko03018	23	0.921282358
Peroxisome	ko04146	21	0.921720576
Histidine metabolism	ko00340	5	0.921794088

Non-homologous end-joining	ko03450	1	0.928988702
Phenylalanine, tyrosine and tryptophan biosynthesis	ko00400	8	0.932433275
Limonene and pinene degradation	ko00903	2	0.933047324
Arginine biosynthesis	ko00220	9	0.943505901
N-Glycan biosynthesis	ko00510	5	0.949532996
Valine, leucine and isoleucine biosynthesis	ko00290	4	0.950058001
Fatty acid degradation	ko00071	11	0.952680915
SNARE interactions in vesicular transport	ko04130	3	0.952732604
One carbon pool by folate	ko00670	3	0.956741835
Pyrimidine metabolism	ko00240	23	0.959573613
Pyruvate metabolism	ko00620	26	0.959885338
Synthesis and degradation of ketone bodies	ko00072	1	0.961090062
Purine metabolism	ko00230	32	0.962984966
ABC transporters	ko02010	10	0.966956421
Alanine, aspartate and glutamate metabolism	ko00250	15	0.971874584
Ubiquitin mediated proteolysis	ko04120	28	0.975291206
Lysine degradation	ko00310	8	0.976024597
Cell cycle - Caulobacter	ko04112	2	0.981943228
Glycine, serine and threonine metabolism	ko00260	14	0.984859202
RNA polymerase	ko03020	3	0.988056891
Oxidative phosphorylation	ko00190	24	0.990342002
Tropane, piperidine and pyridine alkaloid biosynthesis	ko00960	1	0.990822809
Protein processing in endoplasmic reticulum	ko04141	44	0.991332467
Spliceosome	ko03040	34	0.995551747
Fatty acid elongation	ko00062	5	0.995554451
Mismatch repair	ko03430	7	0.998493816
Proteasome	ko03050	5	0.999753256
Citrate cycle (TCA cycle)	ko00020	11	0.999793865
Valine, leucine and isoleucine degradation	ko00280	6	0.999964614

DNA replication	ko03030	4	0.999996695
Homologous recombination	ko03440	4	0.999998953
RNA transport	ko03013	17	0.999999776
Nucleotide excision repair	ko03420	4	0.999999918
Aminoacyl-tRNA biosynthesis	ko00970	3	0.999999996
Ribosome	ko03010	20	1

Table S6. KEGG pathway enriched by DEGs after 12h of cryogenic treatment

Term	ID	DEGs number	P-Value
Photosynthesis - antenna proteins	ko00196	73	1.73E-18
Circadian rhythm - plant	ko04712	41	2.74E-06
Plant hormone signal transduction	ko04075	116	5.20E-05
Phenylpropanoid biosynthesis	ko00940	137	5.52E-05
Starch and sucrose metabolism	ko00500	152	0.0001067
Plant-pathogen interaction	ko04626	128	0.0009885
Phenylalanine metabolism	ko00360	42	0.0011878
Flavonoid biosynthesis	ko00941	27	0.001768
Photosynthesis	ko00195	42	0.0037415
Carotenoid biosynthesis	ko00906	31	0.0039923
Amino sugar and nucleotide sugar metabolism	ko00520	79	0.0044854
Glutathione metabolism	ko00480	69	0.0055412
Pentose phosphate pathway	ko00030	45	0.006204
Glycerophospholipid metabolism	ko00564	64	0.006649
Galactose metabolism	ko00052	49	0.0086473
Stilbenoid, diarylheptanoid and gingerol biosynthesis	ko00945	28	0.0090602
alpha-Linolenic acid metabolism	ko00592	39	0.0096455

Diterpenoid biosynthesis	ko00904	15	0.0117113
Glycerolipid metabolism	ko00561	51	0.0203433
Carbon fixation in photosynthetic organisms	ko00710	58	0.0254722
Pentose and glucuronate interconversions	ko00040	32	0.0443015
Brassinosteroid biosynthesis	ko00905	8	0.0630884
Zeatin biosynthesis	ko00908	14	0.0667974
Arachidonic acid metabolism	ko00590	15	0.068577
Terpenoid backbone biosynthesis	ko00900	34	0.0760563
Glycolysis / Gluconeogenesis	ko00010	88	0.0789687
Ascorbate and aldarate metabolism	ko00053	27	0.0833192
Fructose and mannose metabolism	ko00051	39	0.0978317
Cyanoamino acid metabolism	ko00460	55	0.1051422
Sulfur metabolism	ko00920	27	0.1306993
Monoterpenoid biosynthesis	ko00902	9	0.1311308
Glycosphingolipid biosynthesis - globo series	ko00603	11	0.1346845
Cysteine and methionine metabolism	ko00270	66	0.1437038
Cutin, suberine and wax biosynthesis	ko00073	16	0.1728436
Selenocompound metabolism	ko00450	18	0.1940576
Regulation of autophagy	ko04140	18	0.2085446
Steroid biosynthesis	ko00100	18	0.2547922
Glycosaminoglycan degradation	ko00531	12	0.2612283
Glycosphingolipid biosynthesis - ganglio series	ko00604	7	0.2722579
Arginine and proline metabolism	ko00330	31	0.2748213
Monobactam biosynthesis	ko00261	7	0.3012645
Lysine biosynthesis	ko00300	10	0.3235061
Ether lipid metabolism	ko00565	14	0.3245281
Riboflavin metabolism	ko00740	7	0.3308602
Other glycan degradation	ko00511	13	0.3563142
Folate biosynthesis	ko00790	10	0.4219033

Linoleic acid metabolism	ko00591	13	0.4849376
Vitamin B6 metabolism	ko00750	6	0.5061592
Phenylalanine, tyrosine and tryptophan biosynthesis	ko00400	21	0.5088312
beta-Alanine metabolism	ko00410	21	0.5088312
Nitrogen metabolism	ko00910	22	0.5111298
Biotin metabolism	ko00780	9	0.5169552
Tryptophan metabolism	ko00380	23	0.5290095
Histidine metabolism	ko00340	14	0.5292222
Other types of O-glycan biosynthesis	ko00514	2	0.5307964
C5-Branched dibasic acid metabolism	ko00660	4	0.5339839
Biosynthesis of unsaturated fatty acids	ko01040	16	0.5700439
Ribosome biogenesis in eukaryotes	ko03008	35	0.6084685
Indole alkaloid biosynthesis	ko00901	2	0.6342119
Thiamine metabolism	ko00730	6	0.6777671
Taurine and hypotaurine metabolism	ko00430	6	0.7022698
N-Glycan biosynthesis	ko00510	13	0.7309432
Butanoate metabolism	ko00650	13	0.7309432
Porphyrin and chlorophyll metabolism	ko00860	24	0.7479073
Nicotinate and nicotinamide metabolism	ko00760	15	0.7513855
Ubiquinone and other terpenoid-quinone biosynthesis	ko00130	18	0.7530123
Fatty acid biosynthesis	ko00061	17	0.7566697
Pantothenate and CoA biosynthesis	ko00770	12	0.7812165
Sesquiterpenoid and triterpenoid biosynthesis	ko00909	5	0.7827554
Glycosylphosphatidylinositol(GPI)-anchor biosynthesis	ko00563	9	0.788691
Endocytosis	ko04144	59	0.801417
mRNA surveillance pathway	ko03015	40	0.8166556
One carbon pool by folate	ko00670	8	0.8281154
Purine metabolism	ko00230	62	0.8290744
Limonene and pinene degradation	ko00903	5	0.8378706

Lysine degradation	ko00310	19	0.8549545
Protein export	ko03060	21	0.8783199
Sphingolipid metabolism	ko00600	10	0.8795888
SNARE interactions in vesicular transport	ko04130	7	0.8882451
Inositol phosphate metabolism	ko00562	21	0.8919525
Basal transcription factors	ko03022	17	0.9079224
Tyrosine metabolism	ko00350	14	0.9159352
Peroxisome	ko04146	36	0.919945
Isoquinoline alkaloid biosynthesis	ko00950	5	0.9228767
Non-homologous end-joining	ko03450	2	0.9233394
Caffeine metabolism	ko00232	1	0.9277608
Glyoxylate and dicarboxylate metabolism	ko00630	46	0.9297117
Phagosome	ko04145	23	0.9372195
Pyrimidine metabolism	ko00240	41	0.9428744
ABC transporters	ko02010	19	0.948836
Fatty acid elongation	ko00062	14	0.9511593
RNA polymerase	ko03020	8	0.9519219
Pyruvate metabolism	ko00620	45	0.9579827
Fatty acid degradation	ko00071	19	0.961717
Synthesis and degradation of ketone bodies	ko00072	2	0.9646477
Alanine, aspartate and glutamate metabolism	ko00250	27	0.9672861
Base excision repair	ko03410	10	0.9679531
Glycine, serine and threonine metabolism	ko00260	27	0.9693731
Sulfur relay system	ko04122	2	0.9780418
Ubiquitin mediated proteolysis	ko04120	48	0.9793448
Tropane, piperidine and pyridine alkaloid biosynthesis	ko00960	3	0.9798466
Valine, leucine and isoleucine biosynthesis	ko00290	6	0.9830703
Cell cycle - Caulobacter	ko04112	4	0.9848976
RNA degradation	ko03018	34	0.9854999

Protein processing in endoplasmic reticulum	ko04141	78	0.986168
Arginine biosynthesis	ko00220	13	0.9883578
Mismatch repair	ko03430	17	0.9905705
DNA replication	ko03030	18	0.9977333
Proteasome	ko03050	13	0.9987746
Ribosome	ko03010	76	0.9988835
Oxidative phosphorylation	ko00190	37	0.9990663
Spliceosome	ko03040	52	0.9998114
Homologous recombination	ko03440	16	0.9998223
Citrate cycle (TCA cycle)	ko00020	21	0.9999033
Valine, leucine and isoleucine degradation	ko00280	14	0.9999478
Nucleotide excision repair	ko03420	17	0.9999729
Propanoate metabolism	ko00640	4	0.9999993
RNA transport	ko03013	36	0.9999997
Aminoacyl-tRNA biosynthesis	ko00970	13	0.9999998

Table S7. KEGG pathway enriched by DEGs after 36h of cryogenic treatment

Term	ID	DEGs number	P-Value
Photosynthesis - antenna proteins	ko00196	74	5.66E-09
Circadian rhythm - plant	ko04712	53	0.0002279
Plant-pathogen interaction	ko04626	216	0.0002347
Starch and sucrose metabolism	ko00500	243	0.0002815
Photosynthesis	ko00195	73	0.0004091
Endocytosis	ko04144	155	0.001056
Plant hormone signal transduction	ko04075	172	0.001798
Phenylpropanoid biosynthesis	ko00940	205	0.0022063
Cyanoamino acid metabolism	ko00460	108	0.0035357

Fructose and mannose metabolism	ko00051	76	0.0060051
Glutathione metabolism	ko00480	107	0.0171638
Glycerophospholipid metabolism	ko00564	97	0.0303531
mRNA surveillance pathway	ko03015	100	0.0341853
Ribosome biogenesis in eukaryotes	ko03008	80	0.0435913
Arachidonic acid metabolism	ko00590	25	0.0445306
Pentose phosphate pathway	ko00030	64	0.0505905
Carbon fixation in photosynthetic organisms	ko00710	91	0.0566475
Other types of O-glycan biosynthesis	ko00514	7	0.0723977
Flavonoid biosynthesis	ko00941	31	0.0903139
Glycerolipid metabolism	ko00561	74	0.1252642
Galactose metabolism	ko00052	67	0.1257241
Phagosome	ko04145	65	0.1269857
Protein processing in endoplasmic reticulum	ko04141	191	0.1378362
Ether lipid metabolism	ko00565	27	0.1384603
Steroid biosynthesis	ko00100	32	0.1621938
Amino sugar and nucleotide sugar metabolism	ko00520	108	0.1724471
Inositol phosphate metabolism	ko00562	55	0.1884569
alpha-Linolenic acid metabolism	ko00592	50	0.1885883
Glycosaminoglycan degradation	ko00531	21	0.1891333
N-Glycan biosynthesis	ko00510	32	0.1941034
Regulation of autophagy	ko04140	29	0.2433844
Monobactam biosynthesis	ko00261	12	0.2500536
Diterpenoid biosynthesis	ko00904	15	0.2650036
Monoterpenoid biosynthesis	ko00902	12	0.2836837
Glycosylphosphatidylinositol(GPI)-anchor biosynthesis	ko00563	23	0.3008137
Terpenoid backbone biosynthesis	ko00900	48	0.3113778
Selenocompound metabolism	ko00450	27	0.3344158

Protein export	ko03060	50	0.3548958
Glycolysis / Gluconeogenesis	ko00010	133	0.3649119
Riboflavin metabolism	ko00740	11	0.3778526
Zeatin biosynthesis	ko00908	16	0.3999637
SNARE interactions in vesicular transport	ko04130	20	0.4054461
Basal transcription factors	ko03022	42	0.4241789
Linoleic acid metabolism	ko00591	23	0.4276905
Proteasome	ko03050	50	0.4411621
RNA transport	ko03013	136	0.4564465
Sulfur metabolism	ko00920	37	0.4598384
Cell cycle - Caulobacter	ko04112	18	0.4768688
Carotenoid biosynthesis	ko00906	30	0.4914638
C5-Branched dibasic acid metabolism	ko00660	7	0.4998733
Brassinosteroid biosynthesis	ko00905	7	0.4998733
Stilbenoid, diarylheptanoid and gingerol biosynthesis	ko00945	28	0.5051314
Base excision repair	ko03410	30	0.513982
Cysteine and methionine metabolism	ko00270	98	0.5159218
Ubiquinone and other terpenoid-quinone biosynthesis	ko00130	36	0.5373174
Other glycan degradation	ko00511	19	0.5672985
Phenylalanine metabolism	ko00360	39	0.6045194
Ubiquitin mediated proteolysis	ko04120	108	0.6305167
Pantothenate and CoA biosynthesis	ko00770	24	0.6311079
RNA degradation	ko03018	82	0.6330084
Lysine biosynthesis	ko00300	13	0.637519
Taurine and hypotaurine metabolism	ko00430	11	0.6675424
Ascorbate and aldarate metabolism	ko00053	31	0.6831447
Spliceosome	ko03040	139	0.694002
Sphingolipid metabolism	ko00600	22	0.6952409
Sulfur relay system	ko04122	9	0.7026534

Lipoic acid metabolism	ko00785	3	0.7059249
Indole alkaloid biosynthesis	ko00901	3	0.7059249
Cutin, suberine and wax biosynthesis	ko00073	18	0.7171317
Glycosphingolipid biosynthesis - ganglio series	ko00604	7	0.7447738
Pentose and glucuronate interconversions	ko00040	34	0.7533104
Glycosphingolipid biosynthesis - globo series	ko00603	10	0.7576933
Thiamine metabolism	ko00730	9	0.8133956
Fatty acid biosynthesis	ko00061	29	0.8141482
RNA polymerase	ko03020	19	0.8300232
Caffeine metabolism	ko00232	3	0.8339312
ABC transporters	ko02010	40	0.8422073
Tropane, piperidine and pyridine alkaloid biosynthesis	ko00960	10	0.8486899
Non-homologous end-joining	ko03450	5	0.8530061
Arginine and proline metabolism	ko00330	39	0.8632365
Nicotinate and nicotinamide metabolism	ko00760	24	0.8722903
Pyrimidine metabolism	ko00240	79	0.8750067
Folate biosynthesis	ko00790	11	0.8767297
Peroxisome	ko04146	67	0.8897726
beta-Alanine metabolism	ko00410	28	0.8954789
Phenylalanine, tyrosine and tryptophan biosynthesis	ko00400	28	0.8954789
Purine metabolism	ko00230	106	0.8979113
Biosynthesis of unsaturated fatty acids	ko01040	21	0.9099844
Biotin metabolism	ko00780	10	0.9103978
Sesquiterpenoid and triterpenoid biosynthesis	ko00909	7	0.919735
Lysine degradation	ko00310	32	0.9210322
Tryptophan metabolism	ko00380	30	0.9305561
Porphyrin and chlorophyll metabolism	ko00860	36	0.9399632
Isoquinoline alkaloid biosynthesis	ko00950	9	0.946792

Histidine metabolism	ko00340	16	0.9474789
Vitamin B6 metabolism	ko00750	5	0.9496267
Mismatch repair	ko03430	38	0.9519384
Nitrogen metabolism	ko00910	27	0.951988
Tyrosine metabolism	ko00350	23	0.9704976
Synthesis and degradation of ketone bodies	ko00072	4	0.9736306
Glyoxylate and dicarboxylate metabolism	ko00630	78	0.9752912
Valine, leucine and isoleucine biosynthesis	ko00290	13	0.9756136
One carbon pool by folate	ko00670	10	0.9800945
Propanoate metabolism	ko00640	26	0.9863615
Pyruvate metabolism	ko00620	76	0.9895363
Nucleotide excision repair	ko03420	50	0.9904267
Butanoate metabolism	ko00650	14	0.9917856
Fatty acid elongation	ko00062	22	0.99201
Homologous recombination	ko03440	42	0.992865
Glycine, serine and threonine metabolism	ko00260	44	0.9958758
Limonene and pinene degradation	ko00903	4	0.9964932
Ribosome	ko03010	146	0.9970361
Fatty acid degradation	ko00071	29	0.9970409
Citrate cycle (TCA cycle)	ko00020	51	0.9975708
DNA replication	ko03030	36	0.997659
Oxidative phosphorylation	ko00190	73	0.998451
Valine, leucine and isoleucine degradation	ko00280	37	0.9984593
Arginine biosynthesis	ko00220	17	0.9999253
Alanine, aspartate and glutamate metabolism	ko00250	29	0.9999982
Aminoacyl-tRNA biosynthesis	ko00970	34	0.9999984

Table S8. KEGG pathway enriched by DEGs after 60h of cryogenic treatment

Term	ID	DEGs number	P-Value
Photosynthesis - antenna proteins	ko00196	70	4.95E-09
Endocytosis	ko04144	158	2.39E-05
Cyanoamino acid metabolism	ko00460	110	0.0002259
Starch and sucrose metabolism	ko00500	221	0.0008799
Circadian rhythm - plant	ko04712	46	0.0013007
Phenylpropanoid biosynthesis	ko00940	191	0.002116
Proteasome	ko03050	69	0.0023784
Plant-pathogen interaction	ko04626	187	0.0050192
Plant hormone signal transduction	ko04075	152	0.01011
mRNA surveillance pathway	ko03015	97	0.0144317
Fructose and mannose metabolism	ko00051	66	0.0240909
Amino sugar and nucleotide sugar metabolism	ko00520	108	0.0521571
Protein processing in endoplasmic reticulum	ko04141	185	0.0532579
Galactose metabolism	ko00052	66	0.0591825
Pentose phosphate pathway	ko00030	58	0.0730313
Ether lipid metabolism	ko00565	27	0.079284
Glycerolipid metabolism	ko00561	71	0.0825673
Flavonoid biosynthesis	ko00941	29	0.0879446
Glutathione metabolism	ko00480	91	0.0918354
Phagosome	ko04145	62	0.0931604
Other types of O-glycan biosynthesis	ko00514	6	0.1086017
Base excision repair	ko03410	35	0.1338692
Diterpenoid biosynthesis	ko00904	16	0.1357376
Photosynthesis	ko00195	49	0.1418092
Glycolysis / Gluconeogenesis	ko00010	132	0.1491035
Glycerophospholipid metabolism	ko00564	81	0.1622853

Carbon fixation in photosynthetic organisms	ko00710	78	0.1757346
Arachidonic acid metabolism	ko00590	19	0.1998269
Ubiquinone and other terpenoid-quinone biosynthesis	ko00130	39	0.2292063
SNARE interactions in vesicular transport	ko04130	21	0.2333774
Protein export	ko03060	49	0.238251
alpha-Linolenic acid metabolism	ko00592	45	0.2438774
Glycosaminoglycan degradation	ko00531	18	0.2879295
Taurine and hypotaurine metabolism	ko00430	14	0.2915482
Riboflavin metabolism	ko00740	11	0.3003047
Other glycan degradation	ko00511	21	0.3017737
Indole alkaloid biosynthesis	ko00901	5	0.3078243
Glycosphingolipid biosynthesis - globo series	ko00603	13	0.3796312
Ubiquitin mediated proteolysis	ko04120	106	0.4187493
Steroid biosynthesis	ko00100	25	0.4299543
Glycosphingolipid biosynthesis - ganglio series	ko00604	9	0.4376308
Cell cycle - Caulobacter	ko04112	17	0.449202
Ribosome biogenesis in eukaryotes	ko03008	59	0.4857639
Stilbenoid, diarylheptanoid and gingerol biosynthesis	ko00945	26	0.4990644
Regulation of autophagy	ko04140	23	0.5009836
Sesquiterpenoid and triterpenoid biosynthesis	ko00909	11	0.5128729
Monoterpenoid biosynthesis	ko00902	9	0.5163496
Linoleic acid metabolism	ko00591	20	0.5288434
Carotenoid biosynthesis	ko00906	27	0.5425063
Non-homologous end-joining	ko03450	7	0.5639394
C5-Branched dibasic acid metabolism	ko00660	6	0.5702696
Zeatin biosynthesis	ko00908	13	0.5728509
Sulfur metabolism	ko00920	32	0.5954011
Cutin, suberine and wax biosynthesis	ko00073	18	0.6100039

Pentose and glucuronate interconversions	ko00040	34	0.6102195
Cysteine and methionine metabolism	ko00270	88	0.6113052
Spliceosome	ko03040	131	0.6193144
RNA degradation	ko03018	76	0.6233151
Biotin metabolism	ko00780	13	0.6321306
Pantothenate and CoA biosynthesis	ko00770	22	0.6424341
Lipoic acid metabolism	ko00785	3	0.6622662
Inositol phosphate metabolism	ko00562	41	0.6632592
Glyoxylate and dicarboxylate metabolism	ko00630	87	0.6795517
Terpenoid backbone biosynthesis	ko00900	37	0.7045841
Brassinosteroid biosynthesis	ko00905	5	0.7105189
ABC transporters	ko02010	40	0.7114995
Phenylalanine metabolism	ko00360	34	0.7134076
Glycosylphosphatidylinositol(GPI)-anchor biosynthesis	ko00563	16	0.7155929
One carbon pool by folate	ko00670	15	0.7220585
Sulfur relay system	ko04122	8	0.7330626
Arginine and proline metabolism	ko00330	39	0.7429502
Basal transcription factors	ko03022	33	0.7474174
Selenocompound metabolism	ko00450	19	0.7589014
Vitamin B6 metabolism	ko00750	7	0.775422
Sphingolipid metabolism	ko00600	19	0.7770824
Tryptophan metabolism	ko00380	32	0.7797291
Nicotinate and nicotinamide metabolism	ko00760	24	0.7837483
Caffeine metabolism	ko00232	3	0.798679
Fatty acid biosynthesis	ko00061	27	0.799664
Lysine degradation	ko00310	33	0.8026185
N-Glycan biosynthesis	ko00510	20	0.8047134
beta-Alanine metabolism	ko00410	28	0.809085

Ascorbate and aldarate metabolism	ko00053	26	0.8178025
Monobactam biosynthesis	ko00261	6	0.8181424
Nitrogen metabolism	ko00910	29	0.8303828
Peroxisome	ko04146	64	0.8337602
RNA polymerase	ko03020	17	0.8527992
Butanoate metabolism	ko00650	19	0.8538342
Pyrimidine metabolism	ko00240	73	0.8694566
Lysine biosynthesis	ko00300	9	0.8758365
Folate biosynthesis	ko00790	10	0.8804226
Valine, leucine and isoleucine biosynthesis	ko00290	15	0.891313
Thiamine metabolism	ko00730	7	0.8998007
Purine metabolism	ko00230	97	0.90714
RNA transport	ko03013	108	0.9082625
Citrate cycle (TCA cycle)	ko00020	59	0.9117192
Porphyrin and chlorophyll metabolism	ko00860	34	0.9203514
Limonene and pinene degradation	ko00903	7	0.9254243
Homologous recombination	ko03440	46	0.9259371
Biosynthesis of unsaturated fatty acids	ko01040	18	0.9433971
Mismatch repair	ko03430	35	0.9492891
Tyrosine metabolism	ko00350	22	0.9557781
Synthesis and degradation of ketone bodies	ko00072	4	0.9609584
Fatty acid elongation	ko00062	23	0.9688329
Pyruvate metabolism	ko00620	74	0.9691587
Phenylalanine, tyrosine and tryptophan biosynthesis	ko00400	22	0.9711618
Isoquinoline alkaloid biosynthesis	ko00950	7	0.9756447
Histidine metabolism	ko00340	13	0.9762437
Tropane, piperidine and pyridine alkaloid biosynthesis	ko00960	6	0.9773021
Nucleotide excision repair	ko03420	46	0.9894229

Fatty acid degradation	ko00071	28	0.9935312
Alanine, aspartate and glutamate metabolism	ko00250	41	0.9936031
Glycine, serine and threonine metabolism	ko00260	41	0.9942138
Oxidative phosphorylation	ko00190	70	0.9955681
DNA replication	ko03030	34	0.9959123
Propanoate metabolism	ko00640	21	0.9963085
Valine, leucine and isoleucine degradation	ko00280	35	0.9971361
Arginine biosynthesis	ko00220	18	0.9993917
Aminoacyl-tRNA biosynthesis	ko00970	24	1
Ribosome	ko03010	92	1

Table S9. Real time PCR genes and their primers

Genes		Sequence（5'-3'）
Cluster-37118.46467-F	Sense	GGCTACTAGCTCCCTGGTTG
Cluster-37118.46467-R	Antisense	GTTGGTAGCGCAGCAAGAAG
Cluster-37118.44772-F	Sense	ACATAGCTAGCGCACAGTCC
Cluster-37118.44772-R	Antisense	TGGGTGAATCAGGGAAGCAC
Cluster-37118.46167-F	Sense	TGCTCCAAATCGTCACTGCT
Cluster-37118.46167-R	Antisense	CTGTGCTGTCCACAAGCCTA
Cluster-37118.32850-F	Sense	TGGCAGGACATCAAGAACCC
Cluster-37118.32850-R	Antisense	CGAAGTTGAGCGGGTTGAAG
Cluster-37118.66433-F	Sense	TGTGCAGCTCCCATCATTGT
Cluster-37118.66433-R	Antisense	ACTGTTAAAGACGCCTGGCA
Cluster-37118.39758-F	Sense	TAACTCCTTGCCGATCCGTG
Cluster-37118.39758-R	Antisense	TGCTTATGGAGCTGCTGTCT
